# Neck circumference and its association with cardiometabolic risk factors: a systematic review and meta-analysis

**DOI:** 10.1186/s13098-018-0373-y

**Published:** 2018-09-29

**Authors:** Asal Ataie-Jafari, Nazli Namazi, Shirin Djalalinia, Pouria Chaghamirzayi, Mohammad Esmaeili Abdar, Sara Sarrafi Zadehe, Hamid Asayesh, Maryam Zarei, Armita Mahdavi Gorabi, Morteza Mansourian, Mostafa Qorbani

**Affiliations:** 1 Department of Nutrition, Science and Research Branch, Islamic Azad University, Tehran, Iran; 2 Diabetes Research Center, Endocrinology and Metabolism Clinical Sciences Institute, Tehran University of Medical Sciences, Tehran, Iran; 3 Development of Research & Technology Center, Deputy of Research and Technology, Ministry of Health and Medical Education, Tehran, Iran; 4 Non-communicable Diseases Research Center, Endocrinology and Metabolism Population Sciences Institute, Tehran University of Medical Sciences, Tehran, Iran; 5 Student Research Committee, Alborz University of Medical Science, Karaj, Iran; 6 Non-communicable Diseases Research Center, Alborz University of Medical Sciences, Karaj, Iran; 7 Department of Medical Emergencies, Qom University of Medical Sciences, Qom, Iran; 8 Department of Nutrition and Dietetics, Faculty of Medicine and Health Sciences, Universiti Putra Malaysia, Serdang, Selangor Malaysia; 9 Department of Basic and Clinical Research, Tehran Heart Center, Tehran University of Medical Sciences, Tehran, Iran; 10 Health Management and Economics Research Center, Iran University of Medical Sciences, Tehran, Iran; 11 Chronic Diseases Research Center, Endocrinology and Metabolism Population Sciences Institute, Tehran University of Medical Sciences, Tehran, Iran

**Keywords:** Neck circumference, Metabolic syndrome, Cardiometabolic risk factors

## Abstract

**Background:**

Recently, neck circumference (NC) has been used to predict the risk of cardiometabolic factors. This study aimed to perform a systematic review and meta-analysis to examine: (i) the sensitivity (SE) and specificity (SP) of NC to predict cardiometabolic risk factors and (ii) the association between NC and the risk of cardiometabolic parameters.

**Methods:**

A systematic search was conducted through PubMed/Medline, Institute of Scientific Information, and Scopus, until 2017 based on the search terms of metabolic syndrome (MetS) and cardio metabolic risk factors. Random-effect model was used to perform a meta-analysis and estimate the pooled SE, SP and correlation coefficient (CC).

**Results:**

A total of 41 full texts were selected for systematic review. The pooled SE of greater NC to predict MetS was 65% (95% CI 58, 72) and 77% (95% CI 55, 99) in adult and children, respectively. Additionally, the pooled SP was 66% (95% CI 60, 72) and 66% (95% CI 48, 84) in adult and children, respectively. According to the results of meta-analysis in adults, NC had a positive and significant correlation with fasting blood sugar (FBS) (CC: 0.16, 95% CI 0.13, 0.20), HOMA-IR (0.38, 95% CI 0.25, 0.50), total cholesterol (TC) (0.07 95% CI 0.02, 0.12), triglyceride (TG) concentrations (0.23, 95% CI 0.19, 0.28) and low density lipoprotein cholesterol (LDL-C) (0.14, 95% CI 0.07, 0.22). Among children, NC was positively associated with FBS (CC: 0.12, 95% CI 0.07, 0.16), TG (CC: 0.21, 95% CI 0.17, 0.25), and TC concentrations (CC: 0.07, 95% CI 0.02, 0.12). However, it was not significant for LDL-C.

**Conclusion:**

NC has a good predictive value to identify some cardiometabolic risk factors. There was a positive association between high NC and most cardiometabolic risk factors. However due to high heterogeneity, findings should be declared with caution.

**Electronic supplementary material:**

The online version of this article (10.1186/s13098-018-0373-y) contains supplementary material, which is available to authorized users.

## Background

Cardiovascular diseases are dominant cause of death across the world [1]. Obesity is an important risk factor for these threats and other cardiometabolic diseases such as diabetes [2].

The association between body mass index (BMI), waist circumference (WC), and waist-to-hip ratio (WHR), indices of general or central obesity, with increased cardiometabolic risk has been proved in numerous studies [2, 3]. However, these measures need calibrated tools such as scale, or vary throughout a day. In contrast, neck circumference (NC) is easy to measure, constant, and time-saving measure to identify overweight and obese individuals [4, 5]. It has also been shown as a tool associated with central obesity [6], hypertension and other components of metabolic syndrome (MetS) [7]. A recent meta-analysis from six studies in children and adolescents showed that NC was moderately associated with BMI [8]. To our knowledge, there has been no meta-analysis on sensitivity (SE) and specificity (SP) of NC to identify cardiometabolic risk factors, so far. Moreover, the association between NC and cardiometabolic risk factors has not been examined in child population. Accordingly, we performed a systematic review on studies which assessed NC in association with cardiometabolic risk factors, and studies which reported SE and SP of NC to identify cardiometabolic risk factors.

## Methods

This study was designed as a systematic review on the association of NC and cardio metabolic risk factors. The main related international electronic data sources of PubMed and the NLM Gateway (for MEDLINE), Institute of Scientific Information (ISI), and Scopus searched systematically. For each, strategies were run separately regarding the detailed practical instruction including filters and refining processes. The medical subject headings, Entry Terms and Emtree options were used to reach the most sensitive search.

The strategy developed based on the search terms of MetS, cardio metabolic risk that included all of related components such as glycemic indices including diabetes mellitus, blood glucose, hemoglobin A1c (HbA1c), homeostatic model assessment (HOMA), insulin resistance (IR), lipid profiles including triglycerides (TG), low density lipoprotein (LDL), high density lipoprotein cholesterol (HDL-C), total cholesterol (TC), anthropometric measures including body mass index (BMI), waist circumference (WC), NC, overweight, generalized and abdominal obesity, and blood pressure (BP) including systolic blood pressure (SBP), diastolic blood pressure (DBP), mean arterial pressure (MAP), and their sub-components. At next stage these queries added to results for NC. Data refined for human subject without restriction on language.

We excluded papers of non-population-based studies or those with duplicate citation. For multiple publications of the same population, only the article with largest sample size was included.

The bibliographic information of searched studies saved using Endnote software and four independent reviewers completed all three steps of data refinement, including titles, abstracts and full texts review. Possible disagreements were resolved by third reviewer (M.Qh).

Using Cohen’s kappa statistic, agreement between the results of data extraction of two experts (Sh.D, P.Ch) was 0.94. Data were collected through standard forms which contained author᾽ name, publication year, location, and type of study, sample size, age range, sex, measurements details, and interested outcomes.

### Risk of bias assessment

Risk of bias for studies which reported diagnostic accuracy of NC for predicting cardiometabolic risk factors was assessed using “quality assessment of diagnostic accuracy studies 2” (QUADAS2) checklist. This checklist includes four main methodological domains of study (sample selection, index test, gold standard, process and timing). According to this checklist studies were categorized as “low risk of bias”, “high risk of bias” and “unclear”. The quality assessment of observational studies which assessed association between NC and cardiometabolic risk factors was assessed using the Newcastle–Ottawa checklist which is adapted for types of study (cross sectional, case–control, cohort). In this checklist, each study can attain 9 scores for its quality. Four scores for the selection of study groups, two scores for the comparability of the groups, and three scores for the assessment of outcomes. A study with a Newcastle–Ottawa scale score of ≥ 6 was considered as high quality study. Three authors (H.A, M.Z, A.M) independently evaluated the included studies. A third author (M.M) resolved any disagreements between them.

### Ethical considerations

The protocol of study was approved by the ethical committee of Alborz University of Medical Science. All reviewed studies were properly cited. For more information about a certain study, we contacted the corresponding authors.

### Statistical analysis

The results of diagnostic accuracy of NC to identify MetS was presented as SE, SP and the area under the curve (AUC). The overall (pooled) SE and of SP of NC to identify MetS according to sex and age groups (pediatric and adult) was estimated using random effect meta-analysis method (using the Der-Simonian and Laird method). Forest plot also was used to present result of meta-analysis schematically.

To examine the overall correlation between NC and cardiometabolic risk factors, when r Pearson was reported a mean transformed correlation using r-to-z transformation procedure was used to obtain Fisher’s Z. The standard error was also calculated based on the variance of Fisher’s Z. Spearman was also converted Pearson correlation coefficients, using the following formula:

$${\text{r}}_{{({\text{Pearson}})}} = { 2}*{ \sin }\left( {{\text{r}}_{{({\text{Spearman}})}} *\uppi/ 6} \right)$$

We used Der Simonian and Laird method to pool the correlation coefficients (CC). Between-study heterogeneity was assessed using the I^2^ statistic and I^2^ more than 50% considered as high heterogeneity. Findings were reported separately for adults and children. When the heterogeneity was high, we stratified the studies according to mean age (more or less than 48 years), sex (men, women, both) and continent (Asian, non-Asian) in adult populations. As the range of age in children was similar among the included studies, only sex and continent was considered for subgroup analysis. Stratification was performed when at least two studies were in each sub group. To assess publication bias when there were more than 10 effect sizes, funnel plots and Begg test was used. However, publication bias for variables with less than 10 effect sizes was examined using Egger test. P-value < 0.05 value was considered statistically significant. All statistical analyses were performed with Stata version 12.0 (STATA Corp, College Station, TX, USA).

## Results

**Fig. 1:**
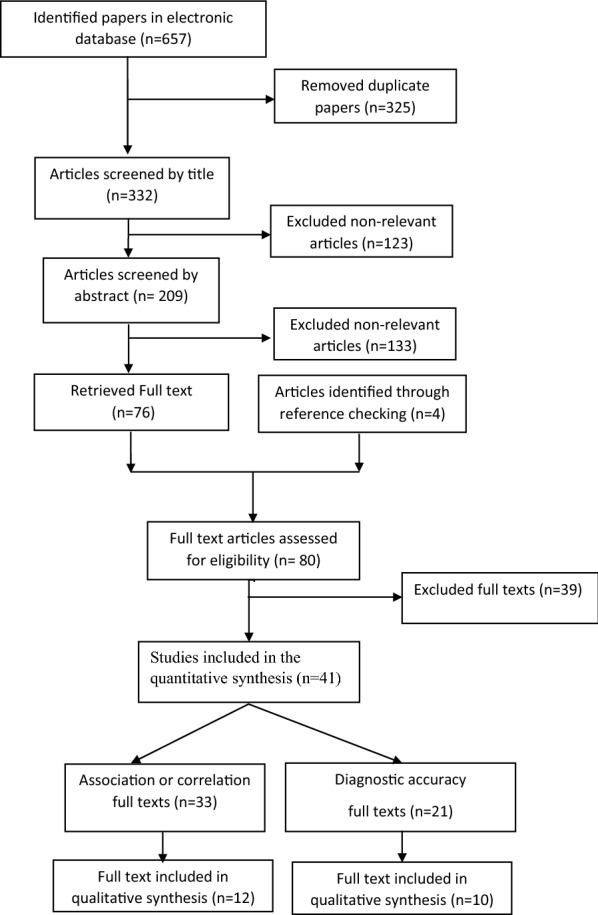
Flowchart of study selection

Figure 1 shows the selection process of articles. In total, 657 records were obtained using searching through PubMed and the NLM Gateway (for MEDLINE), ISI, and Scopus. Subsequently, 325 duplicates were removed. Articles were screened by title and abstract. In addition, 4 articles were identified through reference checking. A total of 80 full texts were assessed for eligibility and finally, 41 articles were selected. The topic of target studies were categorized as follow:

i) Studying diagnostic accuracy of high NC for prediction cardiometabolic risk factors (n = 21).
ii) Studying association between NC and cardiometabolic risk factors (n = 33).

Some studies addressed both of these topics.

### Results of qualitative synthesis

#### A-1: The diagnostic accuracy of high NC to predict cardiometabolic risk factors

A total of 21 articles (including 18 cross-sectional and 3 case–control studies) had reported SE and SP of NC for prediction of cardiometabolic risk factors. They were published between 2010 and 2016 in different countries: China (n = 4), Brazil (n = 3), USA (n = 3), India (n = 3), and 1 article in Colombia, Ukraine, Europe, Turkey, Canada, and Egypt. Eleven studies included children and adolescents and the other 10 ones assessed adults (Table 1).

**Table 1:** Characteristics of the included studies which assessed diagnostic value of high neck circumference to predict cardiometabolic risk factors

Study	Type of study	Country	Target population	Sam pie size	Sex ratio (M/F**)**	Age year	Outco me	Diagnostic criteria	Cut-off values for high NC	Age-sex group	SE % (95% CI)	SP % (95% CI)	AUC (95% CI)
Silva, et al [15]	Cross-sectional	Brazil	Healthy	388	169/219	10–19	Insulin resistance	H0MA1-IR ≥ 3.87 and HOMA1-IR ≤4.19 for females. H0MA1-IR ≥ 3.85 and HOMA1-IR ≥ 3.77 for males	Prepubertal females: > 32.0 cm	Prepubertal females	76.92 (46.2-94.7)	77.50 (61.5–89.1)	0.84 (0.72–0.97)
									Pubertal females: > 34.1 cm	Pubertal females	56.41 (39.6–72.2)	84.75 (77.0–90.7)	0.76 (0.68–0.85)
									Prepubertal males: > 30.3 cm	Prepubertal males	100.00 (78.0–100.0)	42.55 (28.3–57.8)	0.72 (0.58–0.86)
									Pubertal males: > 34.8 cm	Pubertal males	92.00 (73.9–98.8)	57.33 (45.4–68.7)	0.81 (0.71–0.91)
Goncalves et al. [12]	Cross sectional	Brazil	Healthy	260	129/131	10–14	BP	High BP: > 95th percentile	Total:30.2 cm, Male: 30.5 cm. Female: 29.9 cm	Total	80.0	78.4	0.807 (0.754–0.854)
										Female	100.0	69.5	0.908 (0.844–0.951)
										Male	85.7	71.3	0.747 (0.663–0.819)
							TG	Triglycerides ≥ 100 mg/dL		Male	54.6	86.4	0.70 (0.613–0.777)
							HDL-C	HDL-C < 45 mg/dL		Both sexes	61.9	59.3	0.616 (0.554–0.676)
										Female	64.5	64.0	0.645 (0.557–0.727)
							Insulin resistance	Fasting insulin ≥ 15 uU/mL		Both sexes	72.7	57.3	0.703 (0.643–0.757)
										Female	95.7	36.1	0.659 (0.571–0.739); p < 0.05
										Male	100.0	74.0	0.902 (0.837–0.947); p < 0.001
							Diabet es	Fasting glucose ≥ 100 mg/dL		Total	080.0	67.5	0.682 (0.621–0.738); p < 0.001
										Female	100.0	75.8	0.827 (0.751–0.887); p < 0.001
							Excess body fat	Body fat 15–25% for females and 10–20% for males		Total	64.0	65.1	67.6 (0.615–0.733); p < 0.001
										Female	67.6	66.7	0.711 (0.625–0.786); p < 0.001
										Male	75.0	60.7	0.728 (0.643–0.803); p < 0.001
Torriani et al. [37]	Cross-sectiona	USA	Subjects with treated malignancies	303	152/151	18–91	MetS	NCEP Adult Treatment Panel III	43.6 cm in men and 38.6 cm in women	Male	74 (60, 85)	80 (66, 90)	0.79 (0.70, 0.86)
										Female	74 (60, 85)	91 (80, 97)	0.85 (0.76, 0.91)
Formisano et al. [23]	Cross-sectiona	Italy, Belgium, Cyprus, Estonia, Germany, Hungary, Spain and Sweden	Healthy	15673	7962/7711	3–10	CMetS	The components used to calculate cMetS score were the same risk factors used in the adult MetS definition. cMetS score > 90th percentile was considered unfavorable	Male 26.25	Male 3–4 years	47.5	89.5	0.713 (0.622–0.804)
									26.60	4–5 years	58.5	86.4	0.805 (0.740–0.871)
									27.10	5–6 years	82.0	78.6	0.874 (0.820–0.928)
									27.60	6–7 years	83.2	79.9	0.895 (0.856–0.934)
									28.30	7–8 years	79.6	80.3	0.885 (0.848–0.922)
									28.65	8–9 years	88.1	78.0	0.907 (0.872–0.942)
									30.90	9–10 years	71.4	88.1	0.881 (0.772–0.991)
									Female 24.95	Female 3–4 years	63.6	78.6	0.741 (0.662–0.820)
									25.15	4–5 years	81.4	74.4	0.823 (0.764–0.883)
									26.15	5–6 years	75.0	81.0	0.839 (0.772–0.906)
									26.45	6–7 years	94.7	73.8	0.921 (0.884–0.958)
									27.10	7–8 years	88.6	76.3	0.897 (0.862–0.933)
									27.80	8–9 years	93.6	79.0	0.924 (0.898–0.950)
									29.65	9–10 years	1.00	95.0	0.984 (0.955–1.000)
Pillai et al. [27]	Prospective observational cross-sectional	India	Women with PCOS	121	0/121	12–41	MetS	MetS by IDF	33.35	Female	60.3	70.7	0.7 (0.604–0.794)
								MetS by ATP III	33.87	Female	73	69	0.722 (0.631–0.816)
Yan et al. [9]	Cross-sectional	China	Healthy	2092	971/1121	> 65	MetS	The 2004 CDS criteria	38 cm in men and	Male	80	55	0.76
									35 cm in women	Female	75	67	0.73
							Obesity	BMI ≥ 25 kg/m^2^	38 cm in men and 35 cm in women	Male	87	62	NR
										Female	80	74	
Kurtoglu et al. [24]	Case–control	Turkey	Healthy	581	259/322	5–18	MetS	IDF criteria	36 cm in boys and 35 cm in girls	Male	61.9	85.6	0.766 (0.689–0.882)
										Female	60.4	78.15	0.749 (0.683–0.808)
Cizza et al. [38]	Cross-sectional	USA	Obese	120	28/92	18–50	MetS	IDF criteria	NO 38 cm	Female	0.54	0.70	0.63
de LucenaFerretti et al. [20]	Cross-sectional	Brazil	Healthy	1667	751/916	10–17	Overw eight	WHO criteria	> 34.25 in boys a nd > 31.25 ingirls	Male	53.3	72.8	0.690 (0.649–0.730)
										Female	61.2	83.0	0.775 (0.741–0.809)
							Obesity		> 37.95 in boys and > 32.65 ingirls	Male	34.2	94.5	0.712 (0.654–0.770)
										Female	63.8	90.9	0.815 (0.754–0.877)
Yang et al. [10]	Cross-sectional	China	Type 2 diabetic patient	3182	1294/1888	20–80	MetS	Chinese Diabetes Society criteria	NC 39 cm for men and 35 cm for women	Male	42.8	83.8	0.67 (0.63–0.70); p < 0.001
										Female	60.0	66.5	0.66 (0.63–0.70); p < 0.001
							Central obesity	WC ≥ 85 cm for men and ≥ 80 cm for women	NC 37 cm for men and 35 cm for women	Male	65.1	73.6	0.77 (0.72–0.82); p < 0.001
										Female	64.0	75.9	0.75 (0.72–.078); p < 0.001
							Overweight	BMI ≥ 24 kg/m^2^	NC 38 cm for men and 35 cm for women	Male Female	62.0 68.8	74.2 65.4	0.72 (0.69–0.75); p < 0.001 0.73 (0.70–0.75); p < 0.001
Zepeda et al. [39]	Cross-sectional	USA	Healthy	1058	561/497	6–18	High BP	Systolic and/or diastolic BP ≥ 95th percentile for age, sex and height	NC > 90^,th^	Male Female	NR	NR	0.75 (0.70–0.81) 0.72 (0.63–0.75)
Katz et al. [40]	Cross-sectional	Canada	Healthy	1913	977/936	6–17	Overweight/obesity	BMI > 85th percentile CDC	NC > 50th percentile Boys: 25.3–35.5 cm Girls: 24.8–30.5 cm	Total	0.970	0.500	0.884
Lou et al. [41]	Cross-sectional	China	Healthy	2847	1475/1372	7–12	Overweight/obesity	BMI ≥ 85th	Boys: 27.4–31.3 cm Girls: 26.3–31.4 cm	Male Female Total	0.803	0.847	NR
											0.846 0.819	0.843 0.845	
Selvan et al. [13]	Cross-sectional	India	Healthy	451	258/193	30–80	MetS	NCEPATP III	> 34.9 cm for men > 31.25 cm for women	Male Female	78.6 72.3	59.3 64.4	0.753 (0.694–0.813) 0.768 (0.687–0.849
							Type 2 DM		NR	Male Female	NR	NR	0.453 (0.382–0.5324) 0.439 (0.357–0.520)
							Hypertension		NR	Male Female	NR	NR	0.535 (0.465–0.606) 0.501 (0.416–0.586)
							High TG		NR	Male Female	NR	NR	0.670 (0.600–0.739) 0.546 (0.463–0.629)
							Low HDL		NR	Male Female	NR	NR	0.611 (0.537–0.685) 0.622 (0.537–0.707)
Hatipoglu et al. [25]	Case–control	Turkey	Overweight/obese children and healthy ones as control	967	475/492	6–18	Overweight and obesity	BMI ≥ 85th percentile of the BMI reference curve according to local references	Boys: pre-pubertal 29.0 and pubertal 32.5 cm Girls: pre-pubertal 28.0 and pubertal 31.0 cm	Prepubertal male	86.36 (78.5–92.2)	82.58 (75.0–88.6)	0.889 (0.843–0.926)
										Pubertal male	80.85 (71.4–88.2)	76.26 (68.3–83.1)	0.877 (0.828–0.916)
										Prepubertal female	78.95 (68.1–87.5)	85.15 (76.7–91.4)	0.884 (0.828–0.927)
										Pubertal female	83.33 (75.9–89.2)	81.42 (75.0–86.8)	0.896 (0.857–0.928)
Atwa et al. [42]	Cross-sectional	Egypt	Healthy	2762	1327/1435	12–15	Overweight/obesity	BMI > 85th CDC	Men: 29.3–32.3 cm Women:28.6–30.8 cm	Male Female Total	0.927 0.928 0.928	0.806 0.670 0.736	NR
Luo et al. [11]	Cross-sectional	China	Healthy	1943	783/1160	58 ±7	MetS	≥ 2 metabolic disorders But without abdominal obesity	NC > 38.5 cm for men NC > 34.5 cm for women	Male Female	50.53 (45.93–55.12) 48.95 (44.37–53.54)	67.74 (62.23–72.92) 74.85 (71.43–78.07)	NR
							Abdominal obesity	Visceral fatarea of ≥ 80 cm^2^		Male Female	56.1 58.1	83.5 82.5	0.781 0.777
							Diabetes	FPG ≥ 6.10 mmol/Land (or) a 2hPG ≥ 7.80 mmol/L/L, and (or) previously diagnosed diabetes		Male Female	46.33 (41.33–51.38) 43.64 (39.12–48.25)	59.79 (54.73–64.71) 71.08 (67.53–74.44)	NR
							High BP	SBP ≥ 130 mmHg, and (or) DBP ≥ 85 mmHg, and (or) previously diagnosed hypertension serum TG > 1.70 mmol/L		Male Female	48.88 (44.58–53.20) 42.94 (39.17–46.78)	68.98 (62.78– 74.71) 76.18 (72.14–79.90)	NR
							High TG	Serum TG ≥ 1.70 mmol/L		Male Female	52.82 (47.01–58.58) 50.73 (45.29–56.16)	62.66 (58.17–66.99) 71.67 (68.45–74.74)	NR
							Low HDL-C	Serum HDL-C less than 1.04 mmol/L		Male Female	51.45 (44.95–57.92) 58.56 (48.82–67.83)	60.33 (56.07–64.48) 67.59 (64.66–70.42)	
Khalangot et al. [14]	Cross-sectional	Ukraine	Healthy (not registered as T2D patients)	196	46/150	> 44	DM	HbAlc ≥ 6.5%	NO 36.5 cm for women and > 38.5 cm for men	Female	72.2 (46.5–90.3)	62.3 (53.4–70.7)	0.690 (0.815–0.564)
										Male	100 (54.1–100)	38.5 (23.4–55.4)	0.774 (0.682–0.866)
Kumar et al. [26]	Cross-sectional	India	Patients who attended medicine clinic in a tertiary care KMC hospital	431	250/181	Males > 35 and female s > 40	MetS	ATP 111	Total: 36.5cms Female: 34 Male: 37	Total	50.0	76.0	70
										Female	82	32	NR
										Male	68	32	NR
Gomez-Arbelaez et al. [16]	Cross-sectional	Colombia	Healthy	669	351/318	8–14	MetS	Modified NHANES	29 cm in boys and 28.5 cm in girls	Male	100	45.37	0.74 (0.68–0.78)
										Female	87.50	53.61	0.73 (0.68 –0.78)
							Insulin resistance	HOMA-IR22.6	30 cm in males and 29 cm in females	Male	52.54	61.19	0.54 (0.49–0.59)
										Female	50.00	62.35	0.57 (0.51–0.62)

*NC* neck circumference, *HOMA* homeostatic model assessment, *IR* insulin resistance, *HOMA*-*IR* Homeostasis Model Assessment-Insulin Resistance, *HDL*-*C* high density lipoprotein-cholesterol, *NCEP* National Cholesterol Education Program criteria, *cMetS* continuous metabolic syndrome, *CDS* Chinese Diabetes Society, *CDC* Centers for Disease Control and Prevention, *IDF* International Diabetes Federation, *MetS* metabolic syndrome, *BMI* body mass index ,*WHO* World Health Organization, *VFA* visceral fat area, *DM* diabetes mellitus, *FBS* fasting blood glucose, *HbAlc* hemoglobin Ale, *TG* triglycerides, *LDL* low density lipoprotein, *TC* total cholesterol, *WC* waist circumference, *BP* blood pressure, *NR* not reported

The highest SE values of NC for prediction of MetS was 100 in children and 80 in adults. The maximum SP was 89.5 in children and 91 in adults. The SE values to predict overweight/obesity ranged from 34 to 97 in children, and the SP was between 50 and 94. Only 2 studies included adults [9, 10] wherein SE was between 62 and 87 in men, and 68 and 80 in women. SP was between 62 and 74 in men, and between 65 and 74 in women. In 2 studies which reported SE and SP of NC in the prediction of abdominal obesity [10, 11], SE ranged from 56.1 to 68.8 and SP ranged from 65.4 to 83.5. In 2 studies which reported SE and SP of NC for prediction of hypertension among children and adolescents, maximum SE and SP were 85 and 71 in boys and 100 and 69 in girls [12]. Only 3 studies assessed SE and SP of NC for the prediction of high TG and low HDL-C [11–13] wherein the highest values of SE and SP were 62 and 71, respectively.

Among 4 studies which assessed SE and SP of NC for prediction of type 2 diabetes [11–14], the maximum values of SE and SP was 80 and 67 in children [12], and 100 and 72 in adults. In studies which assessed insulin resistance [12, 15, 16], two studies reported a SE of 100 in boys, and 50 to 95 in girls. The SP was 42 to 74 in boys, and 36 to 84 in girls.

According to QUADAS-2 checklist, the study methods of all diagnostic accuracy studies met all QUADAS-2 items. However, three studies were classified as “unclear risk” in the domain of “patient selection” (third question of the first domain) [11, 15, 16]. One studies were classified as “high risk” in the first question of the first domain (random sampling method) [9]. Totally, 83.33% of the studies were considered as high quality (low risk of bias) and 91.66% were classified as low concern according to the QUADAS-2 checklist.

#### A-2: Association between NC and cardiometabolic risk factors

Articles which assessed association between NC and cardiometabolic risk factors were categorized into two sections: articles which assessed cardiometabolic risk factors as binary variables and reported odds ratio (OR) or relative risk (RR) in logistic regression analysis (Table 2), or articles which assessed cardiometabolic risk factors as continuous variables and reported correlation coefficient or Beta coefficient in correlation or linear regression analysis (Table 3).

**Table 2:** Characteristics of the included studies which assessed relationship between neck circumference and cardiometabolic risk factors

Study	**Coun try**	**Type of study**	**Populati on**	**n**	**Male/fe male**	**Agey**	**Diagnostic criteria of outcome**	**Unit of NC**	**Sex group**	**Confounder**	**Outcome**	**Measure of effect**	**Measure of associati on**	**Quality score**
Khalangot et al. [14]	Ukraine	Cross-sectional	Healthy not registere dasT2D patients	196	46/150	> 44	HbAlc ≥ 6.5%		Both sexes	Gender, BMI	DM	1.43 (1.05–1.96); p = 0.024	Adjuste OR (95% CI)	**8**
Yan et al. [9]	China	Cross-sectional	Healthy	2092	971/1121	> 65	According to the 2004 Chinese Diabetes Society (CDS) criteria [1]		Male Female	Age	MetS	11.53 (5.57–23.87) 7.69(4.91-12.04)	Adjusted OR (95% CI) (Q4/Q1)	**7**
							BMI ≥ 25 kg/m^2^		Male Female		Obesity	26.26 (11.02–62.57) 17.16 (9.59–30.70)		
							Fasting TG21.7 mmol/l		Male Female		HighTG	3.06 (2.06–4.54) 2.01 (1.59–2.56)		
							Blood pressure > 140/90 mmHg or known treatment for hypertension		Male Female		HighBP	2.41 (1.94–3.00) 4.37 (2.81–6.7)		
							FBS ≥ 6.1 mmol/l or known treatment for diabetes		Male Female		High FBS	1.89 (1.53–2.34) 1.68 (1.41–2.00)		
Zepeda et al. [39]	USA	Cross-sectional	Healthy	1058	561/497	6–8	Systolic and/or diastolic BP ≥ 95th percentile for age, sex and height	NC > 90th percentile	Both sexes	Age, gender and height	High BP	1.59 (1.05–2.40)	Adjusted OR (95% CI	8
de LucenaFerretti et al. [20]	Brazi1	Cross-sectional	Healthy	1667	751/916	10–17	Overweight and obesity according to the definitions of WHO	≥ 34.25 in boys and ≥ 31.25 in girls	Both sexes	Sex, age, weight, BMI, WC, pubertal stage, SBP, DBP, % body fat	Overweight Obesity	1.70 (0.85–3.39)	AdjustedOR (95% CI	7
								≥ 37.95 in boys and ≥ 32.65 in girls						
												3.26 (1.00–10.59)		
Pereira et al. [17]	Brazi1	Cross-sectional	College students	702	62.7% were women	20–24	NCEP Adult Treatment Panel III	Neck circumference ≥ 39 cm for men and ≥ 35 cm for women	Both sexes	Sex, age, occupational situation	MetS	5.4 (1.4–22.1)	Adjusted OR (95% CI	**7**
Zhou et al. [18]	China	Cross sectional	Healthy	4201	2508/1693	20–85	MetS according to the IDF criteria Increased TG: ( ≥ 1.7 mmol/L) Decreased HDL-C (≤ 1.29 mmol/L for women) High BP:(SBP > 130 orDBP ≥ 85 mmHg) Increased FBS ( ≥ 5.60 mmol/L)	NC of ≥ 37 cm for men and ≥ 33 cm for women	Male	Age, BMI, WC and waist to hip ratio	MetS	1.29 (1.12–1.48)	Adjusted OR (95% CI	**8**
											High BP	1.15 (1.01–1.32)		
											Increased TG	1.16 (1.02–1.33)		
											Increased FBS	1.26 (1.06–1.50)		
									Female		MetS	1.44 (1.20–1.72)		
											High BP	1.22 (1.03–1.46)		
											Increased TG	1.42 (1.18–1.71)		
											Increased FBS	1.32 (1.06–1.65)		
											Decreased HDL-C	1.29 (1.10–1.51)		
Kuciene et al. [22]	Lithuania	Case–control	Case: hypertensive Control: healthy	1947	962/985	12–15	Prehypertension: SBPorDBP ≥ 90th and < 95th percentile Hypertension: SBP or DBP ≥ 95th percentile	NC at > 90th percentile	Both sexes	Age, sex	Prehypertension	2.99 (1.88–4.77)	Adjusted OR (95% CI)	7
											Hypertension	4.05 (3.03–5.41)		
											Prehyper tension/hypertension	3.75 (2.86–4.91)		
Choet al. [19]	South Korea	Cohort	Healthy	3521	1784/1737	42–71	DM was defined based on the WHO criteria	–1st quartile: men: 35.1 cm Women: 30.7 cm –4th quartile: Men: 40.3 cm Women: 35.2 cm	Male Female	Age, BMI orWC, family history of DM, antihypertensive medication, TG, alanine aminotransferase, hsCRP, PRA, HbAlc, HOMA-IRandlGI, daytime sleepiness	Incidence of diabetes mellitus	1.746 (1.037–2.942) 2.077 (1.068–4.038)	Adjusted RR (95% CI**)**	8
Guo et al. [43]	China	Cross-sectional	Normal	6802	3631/3171	5–18	According to The Fourth Report on the Diagnosis, Evaluation, and treatment of High Blood Pressure in Children and Adolescents	NC ≥ 90th percentile	Normal weight subjects	Age, gender BMI, WC	Prehyper tension	1.439 (1.118–1.853)	Adjusted OR (95% CI)	8
									Overweight subjects			1.161 (0.738–1.826)		
									Obese subjects			0.892 (0.429–1.854)		
Vallianou et al. [44]	Greece	Cross-sectional	consecutive adults who had visited the ‘Polykliniki’ General Hospital for a health check-up	490	194/296	18–89	CRP > 0.1 mg/dL		Total	Age and gender years of school, smoking, physical activity status, Diet and alcohol intake	High-SE C-reactive protein	1.14 (1.05–1.23)	Adjusted OR (95% CI)	7
Kelishadi et al. [21]	Iran	Cross-sectional	School students	23043	11708/11335	6–18	Overweight was considered as BMI between the 85th and 94th centiles for age and sex, obesity as BMI ≥ 95th centile; and abdominal obesity as WHtR > 0.5.		Total	Adjusted for age, sex and living area	Over weight General obesity Abdominal obesity	1.07 (1.06–1.08) 1.10 (1.08–1.11) 1.20 (1.18–1.21)	Adjusted OR (95% CI)	7
Zen et al. [45]	Brazi1	Case–control	CHD patients	376	242/134	40 years or over	Significant coronary artery disease defined by the presence of stenosis ≥ 50% in a major epicardial coronary artery-left anterior descendent, circumflex or right coronary artery or their branches with or at least 2.5 mm of diameter	41.6 cm in men and 37.0 cm in women	Total	Age, sex, years at school, smoking, hypertension, HDL-C and diabetes mellitus	Significant coronary stenosis	2.4 (1.1–5.3)	Adjusted OR (95% CI)	7

**Table 3:** Characteristics of the included studies on correlation between neck circumference and cardiometabolic risk factors

Study	Country	Type of study	Population	n	Male/femal e	Agey	Age group	Confounde r	Outcome	Measure of effect	Measure of associati on	Quality score
Kurtoglu et al. [24]	Turkey	Case–contr ol	Healthy	581	259/322	5–18	Prepubertal boys		BMI	r = 0.759; P < 0.001	Pearson correlatio n; P-value	7
									SBP	r = 0.502; P < 0.001		
									DBP	r = 0.335; P < 0.001		
									WC	r = 0.820; P < 0.001		
									FBS	r = 0.172; P = 0.046		
									Insulin	r = 0.609; P < 0.001		
									TC	r = 0.302; P < 0.001		
									TG	r = 0.409; P < 0.001		
									HDL-C	r =− 0.166; P = 0.056		
									HOMA-IR	r = 0.619; P < 0.001		
							Pubertal boys		BMI	r = 0.774; P < 0.001		
									SBP	r = 0.452; P < 0.001		
									DBP	r = 0.472; P < 0.001		
									WC	r = 0.833; P < 0.001		
									FBS	r = 0.047; P = 0.650		
									Insulin	r = 0.325; P < 0.001		
									TC	r = 0.467; P < 0.001		
									TG	r = 0.380; P < 0.001		
									HDL-C	r = − 0.304; P < 0.001		
									HOMA-IR	r = 0.336; P = 0.001		
							Prepubertal girls		BMI	r = 0.783; P < 0.001		
									SBP	r = 0.396; P < 0.001		
									DBP	r = 0.317; P < 0.001		
									WC	r = 0.853; P < 0.001		
									FBS	r = 0.210; P = 0.031		
									Insulin	r = 0.416; P < 0.001		
									TC	r = 0.272; P = 0.005		
									TG	r = 0.208; P = 0.032		
									HDL-C	r = − 0.349; P < 0.001		
									HOMA-IR	r = 0.409; P < 0.001		
							Pubertal Girls		BMI	r = 0.778; P < 0.001		
									SBP	r = 0.268; P < 0.001		
									DBP	r = 0.193; P = 0.008		
									WC	r = 0.781; P < 0.001		
									FBS	r = 0.131; P = 0.074		
									Insulin	r = 0.455; P < 0.001		
									TC	r = 0.101; P = 0.170		
									TG	r = 0.201; P = 0.006		
									HDL-C	r =− 0.189; P = 0.010		
									HOMA-IR	r = 0.449; P < 0.001		
Silva et al. [15]	Brazil	Cross-sectio nal	Healthy	388	169/219	10–19	Male	Body fat percentage and puberty	BMI Z score	0.58; P < 0.001	Adjusted Pearson correlatio n; P-value	6
									WC	0.79; P < 0.001		
									SBP	0.47; P < 0.001		
									DBP	0.37; P < 0.001		
							Female	stage	FBS	− 0.08; P < 0.001		
									Fasting insulin	0.29; P < 0.001		
									HOMA1-IR	0.29; P < 0.001		
									TC	0.08		
									LDL-C	0.14		
									HDL-C	− 0.34; P < 0.001		
									TG	0.23; P < 0.01		
									BMI Z score	0.48; P < 0.001		
									WC	0.64; P < 0.001		
									SBP	0.28; P < 0.001		
									DBP	0.18; P < 0.01		
									FBS	0.08;		
									Fasting insulin	0.43; P < 0.001		
									HOMA1-IR	0.41; P < 0.001		
									TC	0.04;		
									LDL-C	0.09;		
									HDL-C	− 0.24; P < 0.001		
									TG	0.25; P < 0.001		
Goncalves et al. [12]	Brazil	Cross sectio nal	Healthy	260	129/131	10–14	Total		Body fat	0.51; P < 0.001	Pearson correlatio n; P-value	6
									WC	0.74; P < 0.001		
									Weight	0.75; P < 0.001		
									BMI	0.88; P < 0.001		
									Waist to height ratio	0.41; P < 0.001		
									WHR	0.14; P < 0.05		
									HOMA-IR	0.35; P < 0.001		
									Fasting insulin	0.36; P < 0.001		
									SBP	0.62; P < 0.001		
									DBP	0.29; P < 0.001		
									TC	− 0.27; P < 0.001		
									LDL-C	− 0.18; P < 0.05		
									HDL-C	− 0.27; P < 0.001		
									TG	0.06; P < 0.001		
Gomez-Arbelaez et al. [16]	Colombia	Cross-sectio nal	Healthy	669	351/318	8–14	Total	Age, gender and Tanner stage	FBS	0.815 ±0.244; P = 0.001	Adjusted Beta ± SE	7
									HDL-C	− 1.333 ± 0.384; P = 0.001		
									TG	3.887 ± 1.014; P < 0.001		
									SBP	1.719 ±0.205; P < 0.001		
									DBP	1.305 ±0.173;		
										P < 0.001		
									Insulin	0.362 ±0.051; P < 0.001		
									HOMA-IR	0.085 ±0.011; P < 0.001		
Atwa et al. [42]	Egypt	Cross-sectio nal	Healthy	2762	1327/1435	12–15	Male		Weight	r = 0.68; P < 0.001	Pearson correlation coefficient; p-value	8
									BMI	r = 0.67; P < 0.001		
									WC	r=0.72; P < 0.001		
							Female		Weight	r = 0.68; P < 0.001		
									BMI	r = 0.65; P < 0.001		
									WC	r = 0.63; P < 0.001		
Pillai et al. [27]	India	Prospective observational cross-sectional	Women with PCOS	121	0/121	12–41	Female		WC	r = 0.758; p < 0.001	Pearson correlation coefficients	6
Vallianou et al. [44]	Greece	Cross-sectio nal	Consecutive adults who had visited the ‘Polykliniki’ GeneralHos pital for a health check-up	490	194/296	18–89		Age, gender, years of school, smoking, physical activity, diet, alcohol intake	SBP	0.97 (0.41–1.54); p = 0.001	Adjusted Beta (95% CI)	7
									DBP	0.66 (0.31–1.01); P < 0.0001		
									FBS	0.003 (0.001–0.005); p = 0.003		
									HDL-C	_1.37 (_1.77–0.97); p < 0.0001		
									LDL-C	1.15 (_0.05–2.34); p = 0.06		
									TC	1.01 (_0.33–2.35); p = 0.14		
									TG	0.02 (0.01–0.03); p < 0.0001		
Zepeda et al. [39]	USA	Cross-sectional	Healthy	1058	561/497	6–18	Male		WC	r = 0.78; P < 0.001	Pearson correlation coefficients; p-value	8
									BMI	r = 0.72; P < 0.001		
									SBP	r = 0.44; P < 0.001		
									DBP	r = 0.23; P < 0.001		
									WHtR	r = 0.25; P < 0.001		
							Female		WC	r = 0.83; P < 0.001		
									BMI	r = 0.71; P < 0.001		
									SBP	r = 0.41; P < 0.001		
									DBP	r = 0.28; P < 0.001		
									WHtR	r = 0.49; P < 0.001		
Luo et al. [11]	China	Cross-	Healthy	1943	783/1160	58 ±7	Male	Several	Trunk FM	0.444; P < 0.001	Adjusted	8
		sectional						metabolic and body fat parameter s	visceral fat area	0.138; P < 0.001	Beta; p-value	
									Subcutaneous fat area	0.208; P < 0.001		
									SBP	0.052; P = 0.039		
							Female		Trunk FM	0.519; P < 0.001		
									visceral fat area	0.144; P < 0.001		
									Subcutaneous fat area	0.053; P = 0.032		
									SBP	0.098; P < 0.001		
Lou et al. [41]	China	Cross-sectional	Healthy	2847	1475/1372	7–12	Male		Weight	r = 0.841; P < 0.001	Pearson correlation coefficient; p-value	8
									BMI	r = 0.800; P < 0.001		
									WC	r = 0.809; P < 0.001		
							Female		Weight	r = 0.785; P < 0.001		
									BMI	r = 0.736; P < 0.001		
									WC	r = 0.739; P < 0.001		
Selvan et al. [13]	India	Cross-sectio nal	Healthy	451	258/193	30–80	Male	Age	WC	r = 0.742; P < 0.001	Adjusted Pearson correlation coefficient; p-value	
									BMI	r = 0.744; P < 0.001		
									SBP	r = 0.106		
									DBP	r = 0.113		
									FBS	r = 0.025		
									TC	r = 0211; P < 0.05		
									TG	r = 0.365; P < 0.001		
									LDL-C	r = 0.185		
									HDL-C	r = = − 0.319; P < 0.01		
							Female		WC	r = 0.713; P < 0.001		
									BMI	r = 0.682; P < 0.01		
									SBP	r = 0.172		
									DBP	r = 0.028		
									FBS	r = 0.221		
									TC	r= 0.003		
									TG	r = 0.112		
									LDL-C	r = = 0.092		
									HDL-C	r = -0.327; P < 0.01		
Katz et al. [40]	Canada	Cross-sectional	Healthy	1913	977/936	6–17	Healthy-weight male	Age	BMI	0.75 (0.62–0.88)	Adjusted Beta (95% CI)	8
							Overweight/ob ese male		BMI	0.46 (0.38–0.54)		
							Healthy-weight male		WC	0.24 (0.18–0.3)		
							Overweight/ob ese male		WC	0.16 (0.13–0.18)		
							Healthy-weight		BMI	0.42 (0.37–0.47)		
							female					
							Overweight/ob ese female		BMI	0.37 (0.26–0.48)		
							Healthy-weight female		WC	0.15 (0.12–0.17)		
							Overweight/ob ese female		WC	0.15 (0.13–0.17)		
Formisano et al. [23]	Italy, Belgium, Cyprus, Estonia, Germany, Hungary, Spain and Sweden	Cross-sectional	Healthy	15673	7962/7711	3–10	Boys	BMI z-score and country of origin	WC z-score	0.318; P < 0.001	Adjusted Pearson correlation coefficient; p-value	8
									SBPz-score	0.030		
									DBP z-score	− 0.017		
									HDL-C z-score	− 0.060; P < 0.001		
									TG z-score	0.056; P < 0.001		
									HOMA index z-score	0.068; P < 0.001		
							Girls		WC z-score	0.357; P < 0.001		
									SBPz-score	0.050; P < 0.005		
									DBP z-score	− 0.011		
									HDL-C z-score	− 0.056; P < 0.005		
									TG z-score	0.063; P < 0.001		
									HOMA index z-score	0.111; P < 0.001		
Cizza et al. [38]	USA	Cross-sectional	Obese	120	28/92	18–50	Total		MetS score	r = 0.458; p < 0.001	Pearson correlation coefficient; p-value	6
									Fasting insulin	r = 0.476; P < 0.001		
									HOMA index	r = 0.461; P < 0.001		
									Visceral fat	r = 0.674, P < 0.001		
									Subcutaneous fat	r = 0.125, P = 0.20		
									Total abdominal fat%	r = 0.482, P < 0.001		
Yang et al. [10]	China	Cross-sectional	Type 2 diabetic patients	3182	1294/1888	20–80	Male Female		BMI	r = 0.41; P < 0.0001 r = 0.84; P < 0.0001	Pearson correlation coefficient; p-value	8
							Male Female		WC	*r* = 0.47; *P* < 0.0001 r = 0.47; P < 0.0001		
Kumar et al. [26]	India	Cross-sectional	Patients who attended medicine	431	250/181	Males > 35 and females > 40	Total		BMI	0.492; P < 0.001	Pearson correlation coefficient;	7
									WC	0.453; P < 0.001		
									Hip	0.458; P < 0.001		
									W/H RATIO	− 0.005; P = 0.912		
			Clinic in a tertiary care KMC hospital						SBP	0.243; P < 0.001	p-value	
									DBP	0.107; P=0.027		
									FBS	0.166; P < 0.001		
									TC	0.266; P < 0.001		
									LDL	0.344; P < 0.001		
									HDL	− 0.173; P < 0.001		
									TG	0.280; P < 0.001		
Rao et al. [46]	India	Cross-sectional	Patients who visited medicine OPD of a tertiary care teaching hospital	250	180/70	40–100	Total		SBP	0.194056; P = 0.002	Pearson correlation coefficient; p-value	6
									DBP	0.176716; P = 0.005		
Li et al. [47]	China	Cross sectional	Patients who took lower abdomen and neck CT examination s	177	87/90	35–75	Men Women	Age	Visceral adipose tissue (VAT)	r = 0.49, p < 0.00 r = 0.25, p = 0.012	Adjusted Pearson correlation coefficient; p-value	6
							Men Women		Subcutaneous adipose tissue (SAT)	r = 0.59, p < 0.001 r = 0.41, p < 0.001		
Zhou et al. [18]	China	Cross sectional	from the Examination Centre	4201	2508/1693	20–85	Male	Age	SBP	r = 0.250; p < 0.01	Adjusted Pearson correlation coefficient; p-value	8
									DBP	r = 0.261; p < 0.01		
									FBG	r = 0.177; p < 0.01		
									TG	r = 0.240; p < 0.01		
									HDL-C	r = − 0.202; p<0.01		
									TC	r = 0.143; p < 0.01		
									LDL-C	r = 0.088; p < 0.01		
							Female		SBP	r = 0.255; p < 0.01		
									DBP	r = 0.189; p < 0.01		
									FBG	r= 0.180; p < 0.01		
									TG	r = 0.199; p < 0.01		
									HDL-C	r = − 0.234; p<0.01		
									TC	r = 0.039		
									LDL-C	r = 0.075; p < 0.01		
Saka et al. [48]	Turkey	Cross-sectional	Healthy	411	174/237	20–60	Men Women		Body weight	r = 0.576; p=0.000	Pearson correlation coefficient	7
										r = 0.702; p = 0.000		
							Men Women		WC	r = 0.593; p = 0.000		
										r = 0.667; p = 0.000		
							*Men Women		Hip circumferences	r = 0.568; p=0.000 r = 0.617; p = 0.000		
							Men Women		BMI	r=0.58; p = 0.000 r = 0.688; p = 0.000		
Androutsos et al. [28]	Greece.	Cross-sectional	Healthy	324	167/157	9–13	Total	Age, gender, Tanner stage, physical activity, and protein-, carbohydrate- and fat-dietary intake	TC	− 0.200 ± 0.777	Adjusted (β ± SE)	7
									HDL	− 1.713 ± 0.376		
									LDL	1.016 ±0.669		
									Fasting glucose	0.285 ±0.217		
									SBP	2.082 ±0.273		
									DBP	0.465 ±0.234		
									TG	0.037 ±0.009		
									Insulin	0.064 ±0.014		
									HOMA-IR	0.067 ±0.014		
							Male		TC	r = − 0.11	Pearson’s correlation coefficient	
									HDL	r = − 0.32, p<0.001		
									LDL	r = 0.04		
									FBS	r=0.10		
									SBP	r = 0.43, p < 0.001		
									DBP	r = 0.02		
									TG	r = 0.12		
									Insulin	r=0.23, p < 0.001		
									HOMA-IR	r = 0.23, p < 0.001		
							Female		TC	r = − 0.11		
									HDL	r = − 0.23, p<0.001		
									LDL	r = 0.05		
									FBS	r = 0.11		
									SBP	r=0.43, p < 0.001		
									DBP	r = 0.20,p < 0.05		
									TG	r=0.22, p < 0.05		
									Insulin	r = 0.35, p < 0.001		
									HOMA-IR	r = 0.36, p < 0.001		
							Total		TC	r = = − 0.10		
									HDL	r = − 0.27, p<0.001		
									LDL	r = = 0.01		
									FBS	r = 0.11		
									SBP	r = 0.43, p < 0.001		
									DBP	r = 0.09		
									TG	r = 0.15, p < 0.001		
									Insulin	r = 0.26, p < 0.001		
									HOMA-IR	r = 0.26, p < 0.001		
Joshipura et al. [49]	San Juan, USA	Cross-sectional	Overweight/ obese, nondiabetic Hispanics	1206	54.6% male	40–65	Total	Age, gender, smoking status, physical activity	BMI	R = 0.66; p < 0.001	Adjusted Pearson correlation coefficient; p-value	8
									WC	R = 0.64; p < 0.001		
									% body fat	R = 0.45; p < 0.001		
									HOMA-IR	R = 0.45; p < 0.05		
									FBS	R = 0.10; p < 0.001		
									HbAlc	R = 0.28; p < 0.001		
									SBP	R = 0.18; p < 0.001		
									HDL-C	R = − 0.23; p<0.001		
									DBP	R = 0.23;p < 0.001		
									TG	R = 0.12; p < 0.05		
									Hs-CRP	R = 0.30; p <0.001		
Hassan et al. [29]	Egypt	Cross-sectional case control	50 healthy, 50 obese children	100	52/48	7–12	Metabolic subjects		Weight	0.631;P = 0.001	Pearson correlation coefficient; p-value	6
									BMI	0.239; P = 0.240		
									WC	0.465; P = 0.017		
									Waist/Hip	− 0.113; P = 0.582		
									SBP	0.289; P = 0.152		
									DBP	0.445; P = 0.023		
									LDL	0.122; P = 0.551		
									HDL	− 0.120; P = 0.559		
									TC	0.056;P = 0.787		
									TG	− 0.253; P = 0.212		
									FBS	− 0.377; P = 0.058		
									Fasting Insulin	0.219; P = 0.283		
									HOMA-IR	0.113;P = 0.583		
							Non metabolic subjects		Weight	0.619; P = 0.001		
									BMI	0.535;P = 0.007		
									WC	0.605; P = 0.002		
									Waist/Hip	− 0.203; P = 0.340		
									SBP	0.048; P = 0.823		
									DBP	0.186; P = 0.384		
									LDL	− 0.444; P = 0.030		
									HDL	− 0.139; P = 0.516		
									TC	− 0.221; P = 0.299		
									TG	0.314; P = 0.135		
									FBS	− 0.137; P = 0.524		
									Fasting Insulin	0.119; P = 0.580		
									HOMA-IR	0.116; P = 0.591		
Cho et al.[19]	South	Conor	Healthy	3521	1784/1737	42–71	Male		SBP	0.170; P < 0.001	Pearson	8
	Korea	t							DBP	0.200; P < 0.001	Correlation coefficient; p-value	
									BMI	0.801; P < 0.001		
									WC	0.740; P < 0.001		
									Body fat (%)	0.547; P < 0.001		
									FPG	0.159; P < 0.001		
									HOMA-IR	0.317; P < 0.001		
									TG	0.240; P < 0.001		
									HDL-C	− 0.246; P < 0.001		
							Female		SBP	0.203; P < 0.001		
									DBP	0.199; P < 0.001		
									BMI	0.744; P < 0.001		
									WC	0.706; P < 0.001		
									Body fat (%)	0.510; P < 0.001		
									FPG	0.122; P < 0.001		
									HOMA-IR	0.234; P < 0.001		
									TG	0.256; P < 0.001		
									HDL-C	− 0.223; P < 0.001		
Guo et al. [43]	China	Cross-sectio nal	Normal	6802	3631/3171	5–18	Normal weight	Age, gender, BMI, WC	BMI	r = 0.226; P < 0.001	Adjusted Pearson correlation coefficient; p-value	8
									WC	r = 0.339; P < 0.001		
									SBP	r = 0.449; P < 0.001		
									DBP	r = 0.328; P < 0.001		
							Overweight		BMI	r = 0.137; P < 0.001		
									WC	r = 0.348; P < 0.001		
									SBP	r = 0.459; P < 0.001		
									DBP	r = 0.344; P < 0.001		
							Obese		BMI	r = − 0.004;P = 0.932		
									WC	r = 0.635; P < 0.001		
									SBP	r = 0.477; P < 0.001		
									DBP	r = 0.325; P < 0.001		
Hatipoglu et al. [25]	Turkey	Case–control	Overweight/ obese children and healthy ones as control	967	475/492	6–18	Boys prepubertal pubertal Girls prepubertal pubertal		BMI	r = 0.700; P < 0.001 r = 0.821; P < 0.001 r = 0.727; P < 0.001 r = 0.848; P<0.001	Pearson correlation coefficient; p-value	8
							Boys Prepubertal Pubertal Girls Prepubertal Pubertal		WC	r = 0.733; P < 0.001 r = 0.839; P < 0.001 r = 0.776; P < 0.001 r = 0.854; P<0.001		
Kelishadi et al. [21]	Iran	Cross-	School	23043	11708/113	6–18	Male	Age, sex	Weight	r = 0.546; p < 0.001	Adjusted	7
		sectional	students		35			and living area	BMI	r = 0.389; p < 0.001	Pearson correlation coefficient; p-value	
									WC	r = 0.491; p < 0.001		
									Waist/Hip	r = 0.035; p < 0.001		
									Waist/Height	r = 0.156; p < 0.001		
									Hip	r = 0.505; p < 0.001		
							Female		Weight	r = 0.481; p < 0.001		
									BMI	r = 0.387; p < 0.001		
									WC	r = 0.456; p < 0.001		
									Waist/Hip	r = − 0.020; p < 0.001		
									Waist/Height	r = 0.222; p < 0.001		
									Hip	r = 0.464; p < 0.001		
							Total		Weight	r = 0.519; p < 0.001		
									BMI	r = 0.384; p < 0.001		
									WC	r = 0.479; p < 0.001		
									Waist/Hip	r = 0.023; p < 0.001		
									Waist/Height	r = 0.188; p < 0.001		
									Hip	r = 0.478; p < 0.001		

Table 2 lists characteristics of studies reporting OR/RR of high NC and the risk of cardiometabolic risk factors (n = 13). Most of them were designed as cross-sectional (n = 10) and the rest as case–control (n = 2) or cohort (n = 1). The studies were carried out in different countries including China (n = 3), Brazil (n = 3), Greece (n = 2), and one study in Ukraine, USA, Iran, Lithuania, and South Korea. In 6 studies, children and adolescents were included, and 7 reports were on adult populations. The articles have been published between 2012 and 2017.

Three studies in adults assessed the OR of high NC in prediction of MetS presence [9, 17, 18]. Among them, Yan et al. found the strongest association between high NC and MetS in both elderly men and women, with ORs of 11.53 and 7.69, respectively [9]. The association between high NC and DM was reported in few studies [9, 14, 18, 19] where in ORs or RRs varied between 1.26 (1.06–1.50) and 2.07 (1.06–4.03).

Three studied reported the association between high NC and obesity. Among children and adolescents, ORs was between 1.07 and 1.70 for the prediction of overweight, and 1.10 to 3.25 for prediction of obesity [20, 21].Yan et al. found again a strong association between high NC and obesity among elderly men and women, with ORs of 26.26 and 17.16, respectively [9].

In two studies which assessed the association between high NC and high TG [9, 18], the ORs were between 1.16 and 3.06. In regard to high BP, Kuciene et al. [22] found that greater NC was associated with 4 times risk for hypertension. Among adults, Yan et al. [9] found OR of 2.41 and 4.37 in elderly men and women, respectively.

Table 3 shows association studies where both NC and cardiometabolic risk factors were reported continuous variables. A total of 27 studies were found (14 publications included children and adolescents, and 13 studies in adults). Most of them used correlation coefficients, and few ones used beta regression coefficients for statistical analyses. The articles were published between 2010 and 2017. The studies were carried out in different countries including China (n = 6), India (n = 4), USA (n = 3), Turkey (n = 3), Brazil (n = 2), Egypt (n = 2), Greece (n = 2), and one study in Iran, Canada, Europe, Colombia, and South Korea.

Out of 18 studies which assessed the correlation between NC and BMI, 11 articles included children and adolescents. Significant correlations were found between NC and BMI. The *r* ranged from 0.38 [21] to 0.88 [12] in adolescents. In adults, *r* ranged from 0.41 to 0.84 in men and women together.

There was a significant association between WC and NC in all 20 studies (13 reports in children and adolescents, and 8 studies in adults). The *r* ranged from 0.318 [23] to 0.85 [24, 25] among children and adolescents. In adults, *r*-values was between 0.45 [26] and 0.75 [27].

Out of 18 studies which reported the correlation between NC and blood pressure, 9 publications were on children and adolescents. A wide range of *r* was found; from 0.02 [28] to 0.62 [12]. In some studies, the correlation was not significant [13, 28, 29].

Weak correlations was observed between NC and FBS in 12 relevant studies, (*r* ranged from − 0.377 to 0.27 [29, 30]). Eleven studies also reported correlation between fasting insulin, HOMA-IR or both with NC. The *r*-values for these two variables were very close, ranging from 0.21 to 0.61 [24, 30].

Fourteen studies reported correlation coefficients of blood TC, TG, HDL-C, or LDL-C with NC. Findings of correlation between TC and NC was not conclusive; *r*-values ranged from − 0.27 [12] to 0.302 [24]. Blood TG was positively correlated with NC in all reports [*r* ranged from 0.06 [12] to 0.409 [24]. There was negative correlation between HDL-C and NC in all relevant publications, with *r* ranging from − 0.120 [29] to − 0.35 [30]. Weak and mostly not significant correlations between LDL-C and NC were observed.

According to the Newcastle–Ottawa checklist, all selected studies were categorized as high quality study and attained score ≥ 6 according to this scale. Overall, 20% of studies attained 6 scores, 38% of studies attained 7 scores and the rest got the score of 8 (Tables 2 and 3).

### Results of quantitative synthesis

#### B-1: The diagnostic accuracy of high NC to predict MetS

The results of heterogeneity statistics about the SE of high NC to predict MetS according to sex and age groups showed sever heterogeneity in SE existed between studies in male (I^2^: 97.9%; Q test: 335.85, p < 0.001), female (I^2^: 91.1%; Q test: 112.26, p < 0.001), pediatric (I^2^: 91.1%; Q test: 33.75, p < 0.001), adult (I^2^: 96.2%; Q test: 391.78, p < 0.001), and overall population (I^2^: 96%; Q test: 479.02, p < 0.001). Due to sever heterogeneity between studies, the random effect meta-analysis was used and the pooled SE in male, female, pediatric, adult and overall population was estimated 69% (95% CI 56–83), 67% (95% CI 60–74), 77% (95% CI 55–99), 65% (95% CI 58–72) and 67% (95% CI 61–74), respectively (Additional file 1: Figure S1:A–D). The results of heterogeneity statistics for SP of high NC to predict MetS indicated sever heterogeneity among studies in both sexes and age groups. The random effect meta-analysis showed that the pooled SP in male, female, adult, pediatric and overall population was 64% (95% CI 52, 75), 67% (95% CI 60, 74),66% (95% CI 60, 72), 66% (95% CI 48, 84) and 66% (95% CI 60, 73), respectively (Additional file 2: Figure S2: E–H).

Publication bias: Begg᾽s test confirmed no publication bias for sensitivity (p = 0.32) and specificity (p = 0.92) of high NC for predicting MetS.

#### B-2: The association of NC with glycemic indices in adult populations

FBS: The pooled estimates of 4 studies (seven effect sizes) indicated that there was a significant positive correlation between NC and serum levels of FBS (CC: 0.16, 95% CI 0.13, 0.20). However, the heterogeneity was high (I^2^: 56.0%, p = 0.03) (Additional file 3: Figure S3:1). Subgroup analysis based on age, sex and continent are presented in Additional file 4: Table S1. After stratification by continent (Asian, Non-Asian), we found that the association between NC and FBS concentrations in Asian population (CC: 0.19, 95% CI 0.16, 0.22; I^2^:0, p = 0.61) was stronger than Non-Asian (CC: 0.13, 95% CI 0.10, 0.16; I^2^: 28.3%, p = 0.24). This parameter attenuated the heterogeneity greater than gender subgroups.

HOMA-IR: The association between NC and HOMA-IR was reported in three studies containing four effect sizes. The overall effect size showed a significant link between NC and HOMA-IR (CC: 0.38, 95% CI 0.25, 0.50) in adult population, while the heterogeneity was high (I^2^: 93.5%, p = 0.0001) (Additional file 3: Figure S3:2). Due to limited studies, it was not possible to perform subgroup analysis to find the reason of the heterogeneity.

#### B-3: The association of NC with lipid profile in adult populations

Based on the overall effect size, in subjects who had higher NC, serum levels of TC was higher than those with smaller one (CC: 0.12, 95% CI 0.05, 0.19; I^2^: 79.2, p = 0.001) (Additional file 3: Figure S3:3). After stratification by age, a notable reduction was observed in the heterogeneity (Additional file 4: Table S1). Besides, pooling 8 effect sizes revealed that there was a significant correlation between NC and TG concentrations (CC: 0.23, 95% CI 0.19, 0.28; I^2^: 76.2%, p = 0.001) (Additional file 3: Figure S3:4). However, after subgroup analysis the heterogeneity did not attenuate considerably (Additional file 4: Table S1). Meta-analysis on LDL-C concentrations also showed a positive association with NC (CC: 0.14, 95% CI 0.07, 0.22); however, the heterogeneity was high (I^2^: 79.2%, p = 0.001) (Additional file 3: Figure S3:5). Subgroup analysis showed that this association in men (CC: 0.13, 95% CI 0.03, 0.22; I^2^: 59.1%, p = 0.11) was stronger than women (CC: 0.08, 95% CI 0.03, 0.13; I^2^: 0%, p = 0.81).

Publication bias: Egger᾽s test showed no publication bias for FBS (p = 0.49), HOMA-IR (p = 0.57), TC (p = 0.92), TG (p = 0.93) and LDL-C (p = 0.25).

#### B-4: The association of NC with glycemic indices in child populations

FBS: From five studies in which the association between NC and FBS concentrations was reported, 12 effect sizes were extracted. The pooled estimates showed that children with greater NC had higher levels of FBS compared to those with smaller one (CC: 0.12, 95% CI 0.07, 0.16; I^2^:48.4%, p = 0.03) (Additional file 5: Figure S4:1). No severe heterogeneity was found for this association.

HOMA-IR: The correlation between NC and HOMA-IR was reported in 6 studies including 11 effect sizes. Based on findings, greater NC was correlated with higher HOMA-IR (CC: 0.27, 95% CI 0.23, 0.31). However, the heterogeneity was considerably high (I^2^: 93.2%, p = 0.0001) (Additional file 5: Figure S4:2).

#### B-5: The association of NC with lipid profile in child populations

The pooled estimates (n = 12) of five studies showed a significant positive link between NC and TC concentrations (CC: 0.07 95% CI 0.02, 0.12; I^2^:87.8%, p = 0.001), although it was a weak correlation (Additional file 5: Figure S4:3). Findings of six studies also revealed a significant link between NC and TG levels (CC: 0.21, 95% CI 0.17, 0.25; I^2^:61.2%, p = 0.001) (Additional file 5: Figure S4:4). However, no correlation was obtained between NC and LDL-C (CC: 0.01, 95% CI − 0.06, 0.07; I^2^:65.9%, p = 0.005) (Additional file 5: Figure S4:5). Due to limited studies on children, subgroup analyses were not possible.

Publication bias: Begg᾽s test confirmed no publication bias for FBS (p = 0.19), HOMA-IR (p = 0.38), TC (p = 0.37), TG (p = 0.58) and LDL-C (p = 0.06).

## Discussion

The current systematic review and meta-analysis revealed a positive association of NC, glycemic status and lipid profile in adult and child populations. However, no correlation was observed between NC and LDL-C concentrations in children. In general, due to high heterogeneity the findings should be declared with caution. Moreover, the association between NC and other cardio-metabolic risk factors were significant in most studies. However, because of limited studies drawing a certain decision needs further studies. Although the SE and the SP of NC to predict MetS were greater than about 65% in both child and adult populations, the between-study heterogeneity was considerably high.

To the best of our knowledge, the present study is the first study that examined the association of NC and cardio-metabolic risk factors in all age ranges and determined the SE and the SP of NC to predict MetS. In the present study, subgroup analysis revealed that the link between serum levels of FBS and NC in Asian was stronger than other adult populations. Findings on children populations also showed that the link between NC and FBS was significant only in Asian populations. Additionally, in Asian children the link between insulin resistance and NC was stronger than non-Asians.

These findings showed that race can play a main role in this correlation. Besides, the correlation between NC and LDL-C levels in men was stronger than the correlation in adult women. Therefore, gender can be another factor that affects the association. Energy intake, physical activity level, and menopause status are possible factors that can affect the link. In the present study, some included studies did not control such factors and it is likely to cause bias in the findings.

Another factor in the association between NC and cardio-metabolic risk factors is likely to be study design. In the present systematic review, design in most studies was cross-section. The weakness of this kind of study is inability to clarify a cause and effect relationship. Prospective cohort studies can shed light on the type of the association.

Although prior studies introduced WC as a good predictor for cardio-metabolic risks [31, 32], it has some limitations. For instance, several sites including midway between rib cage and iliac crest, the lower border of rib cage, and iliac crest umbilicus are used for measuring WC. This resulted in different values for WC. Moreover, time of measurement, the state of expiration and fullness affect the measure [29, 33]. However, NC measurement is easy and accessible. Besides, a unit site for measurement was reported among the studies. NC is measured above the cricoid cartilage and perpendicular to the long axis of the neck [34, 35]. Due to no variation in the measurement of NC, multiple measurements are not needed to be sure about its accuracy.

Compare to BMI, NC has some strength points. NC is measured faster and does not need special tools [9]. Therefore, particularly for epidemiological assessment it seems to be a good predictor. However, due to the high heterogeneity, more studies are needed to clarify its efficacy.

In the present study, we found that the association of NC with obesity, diabetes, hypertension, and MetS were significant in most studies. However, due to limited studies we cannot draw a fix conclusion about these issues. In addition, as there has been no meta-analysis on the SE and the SP of NC as a predictor for MetS, we could not compare our results with previous findings. Based on a systematic review by Arias et al., there was a positive association between NC and adiposity parameters indirectly measured by reference methods including dual-energy x-ray absorptiometry (DXA) and computed tomography (CT) in adult population. However, they reported no study on children in this regard [50].

The mechanisms that explain the association between neck adipose tissue and cardio-metabolic risk factors are not precisely identified. It is likely that high plasma free fatty acids (FFAs) provide a ground for developing metabolic disorders [36]. Increasing in the levels of FFAs can result in oxidative stress and vascular injury [15, 36]. The main releasing rate of systemic FFA is dedicated to upper body subcutaneous fat [5, 36]. Accordingly, NC can be a suitable predictor for CVD risk factors.

The present study has two main limitations: [1] due to cross-sectional design in the most included studies a cause and effect relationship was not clarified. [2] Heterogeneity mostly remained high even after stratification by possible confounders. The main strength of the present systematic review was to determine the SE and SP of NC in adult and child populations.

## Conclusion

Although the SE and the SP of NC to predict MetS were acceptable in both child and adult population, the between-study heterogeneity was considerably high. There is a positive association between NC and glycemic indices, and lipid profile in adult and pre-pubertal populations. However, no correlation was observed between NC and LDL-C concentrations in children. Due to high heterogeneity, the findings should be declared with caution. Although the association between NC and other cardio-metabolic risk factors were significant in most studies, due to limited publications in this regard more prospective cohort studies are needed to clarify these associations.

## Additional files

**Additional file 1: Figure S1.** Forest plot of high neck circumference sensitivity for predicting metabolic syndrome in A) male, B) female, C) children, D) adult population.

**Additional file 2: Figure S2.** Forest plot of high neck circumference specificity for predicting metabolic syndrome in E) male, F) female, G) children, H) adult population.

**Additional file 3: Figure S3.** Forest plot of the association of neck circumference and 1) FBS, 2) HOMA, 3) TC, 4) TG, 5) LDL-C in adult population.

**Additional file 4: Table S1.** Subgroup analysis for the association between neck circumference and cardio-metabolicfactors in adult population.

**Additional file 5: Figure S4.** Forest plot of the association of neck circumference and 1) FBS, 2) HOMA, 3) TC, 4) TG, 5) LDL-C in child population.
