# Supplementary material for: Neck circumference and its association with cardiometabolic risk factors: a systematic review and meta-analysis
Source: Diabetol Metab Syndr. 2018 Sep 29;10:72. doi: 10.1186/s13098-018-0373-y (PMC6162928; doi:10.1186/s13098-018-0373-y)
Supplement: Supplementary file 2 — Additional file 2: Figure S2. Forest plot of high neck circumference specificity for predicting metabolic syndrome in E) male, F) female, G) children, H) adult population. [file 13098_2018_373_MOESM2_ESM.docx]

E

F

G

H
