# Supplementary figures and images for: Neck circumference and its association with cardiometabolic risk factors: a systematic review and meta-analysis
Source: Diabetol Metab Syndr. 2018 Sep 29;10:72. doi: 10.1186/s13098-018-0373-y (PMC6162928; doi:10.1186/s13098-018-0373-y)

| Study |             |  |  | %      |
|-------|-------------|--|--|--------|
| ID    | ES (95% CI) |  |  | Weight |

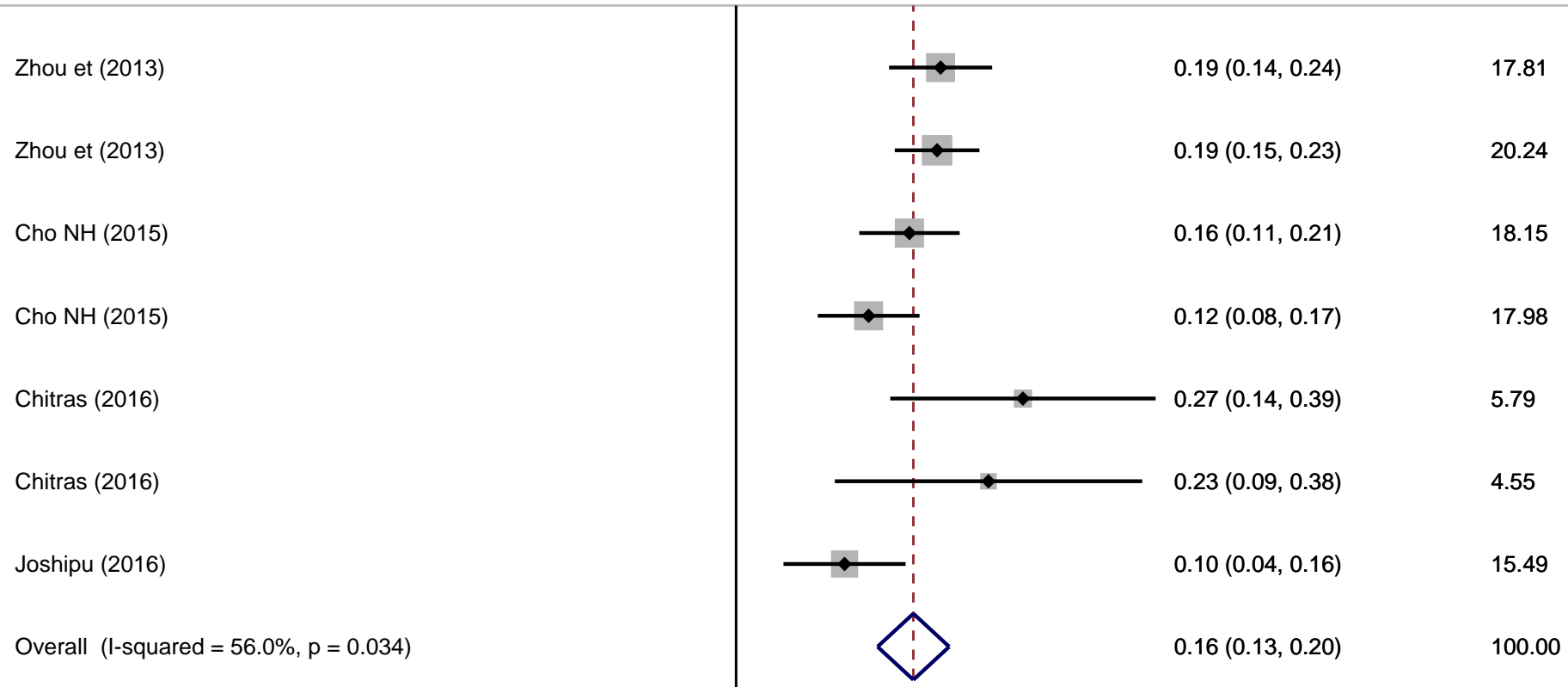

NOTE: Weights are from random effects analysis

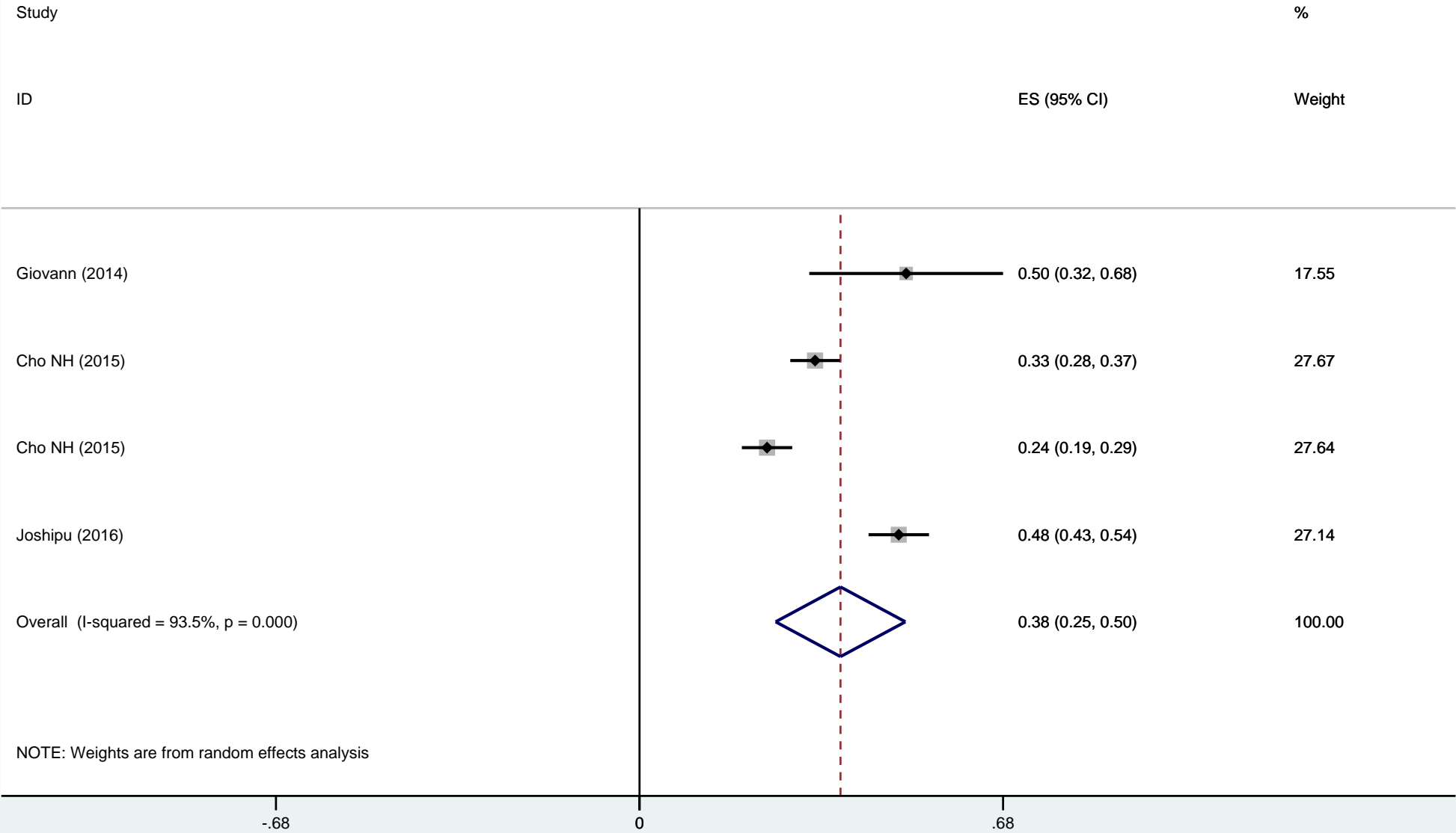

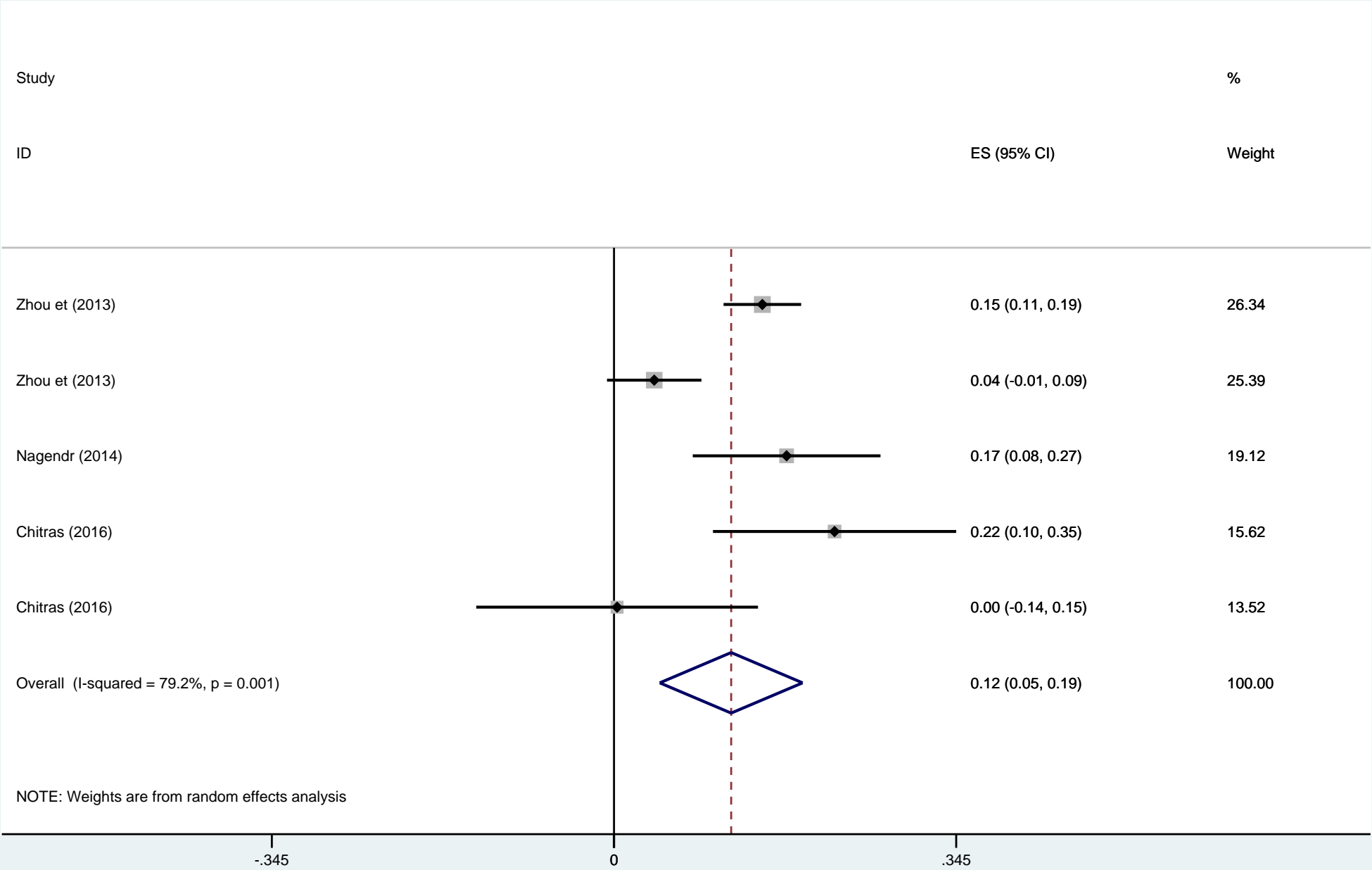

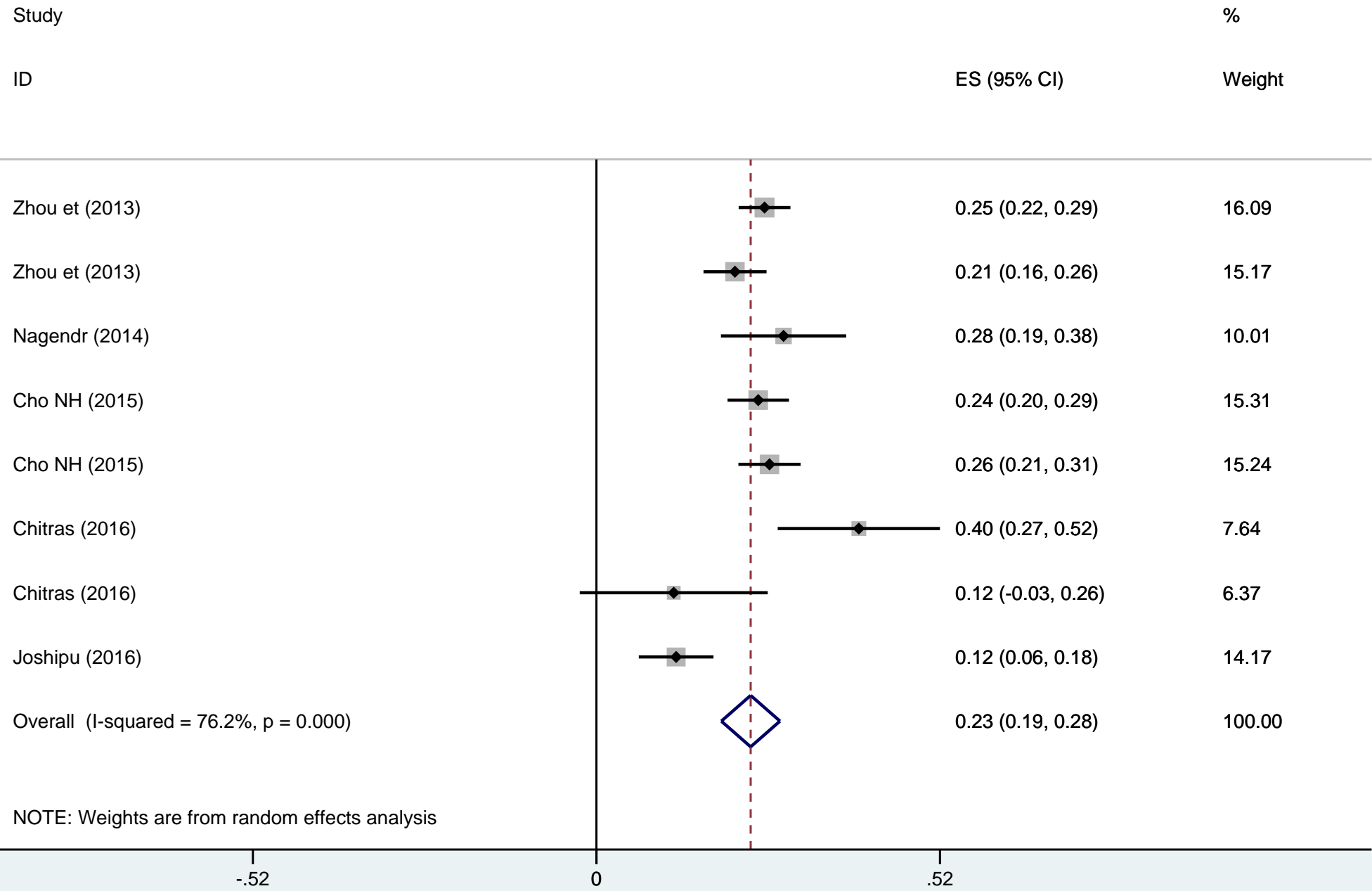

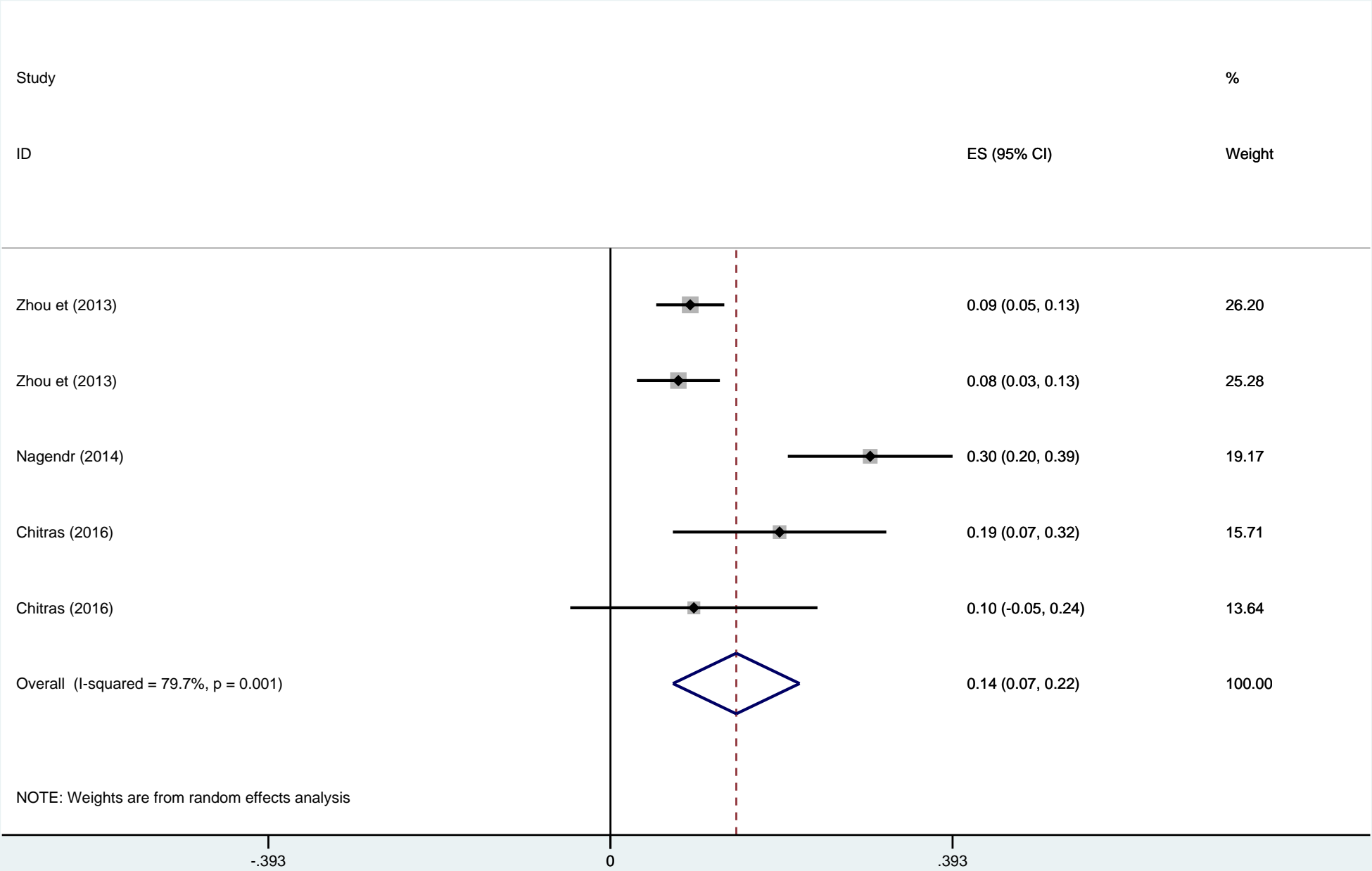

Supplement: Supplementary file 3 — Additional file 3: Figure S3. Forest plot of the association of neck circumference and 1) FBS, 2) HOMA, 3) TC, 4) TG, 5) LDL-C in adult population. [file 13098_2018_373_MOESM3_ESM.pdf]

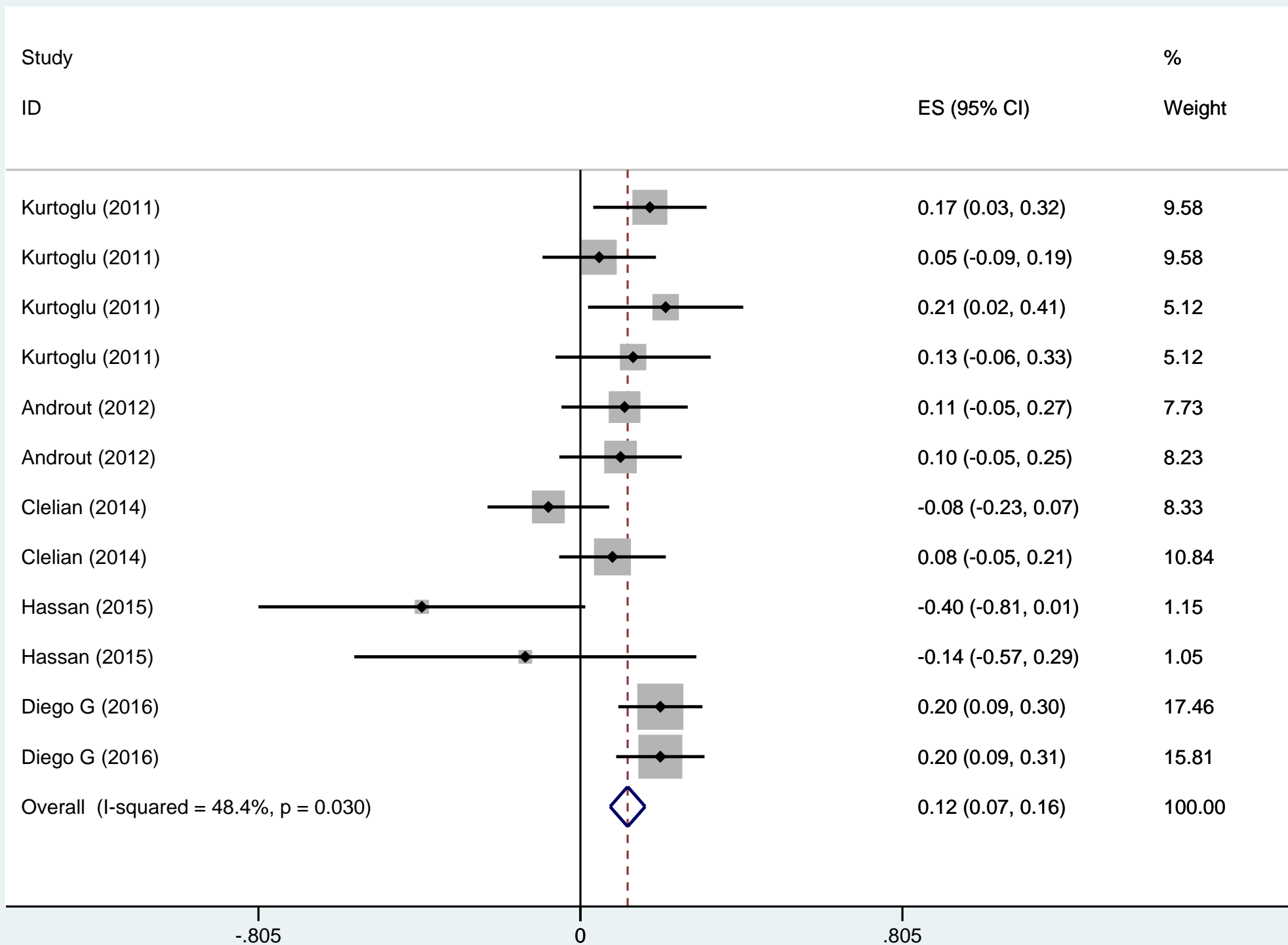

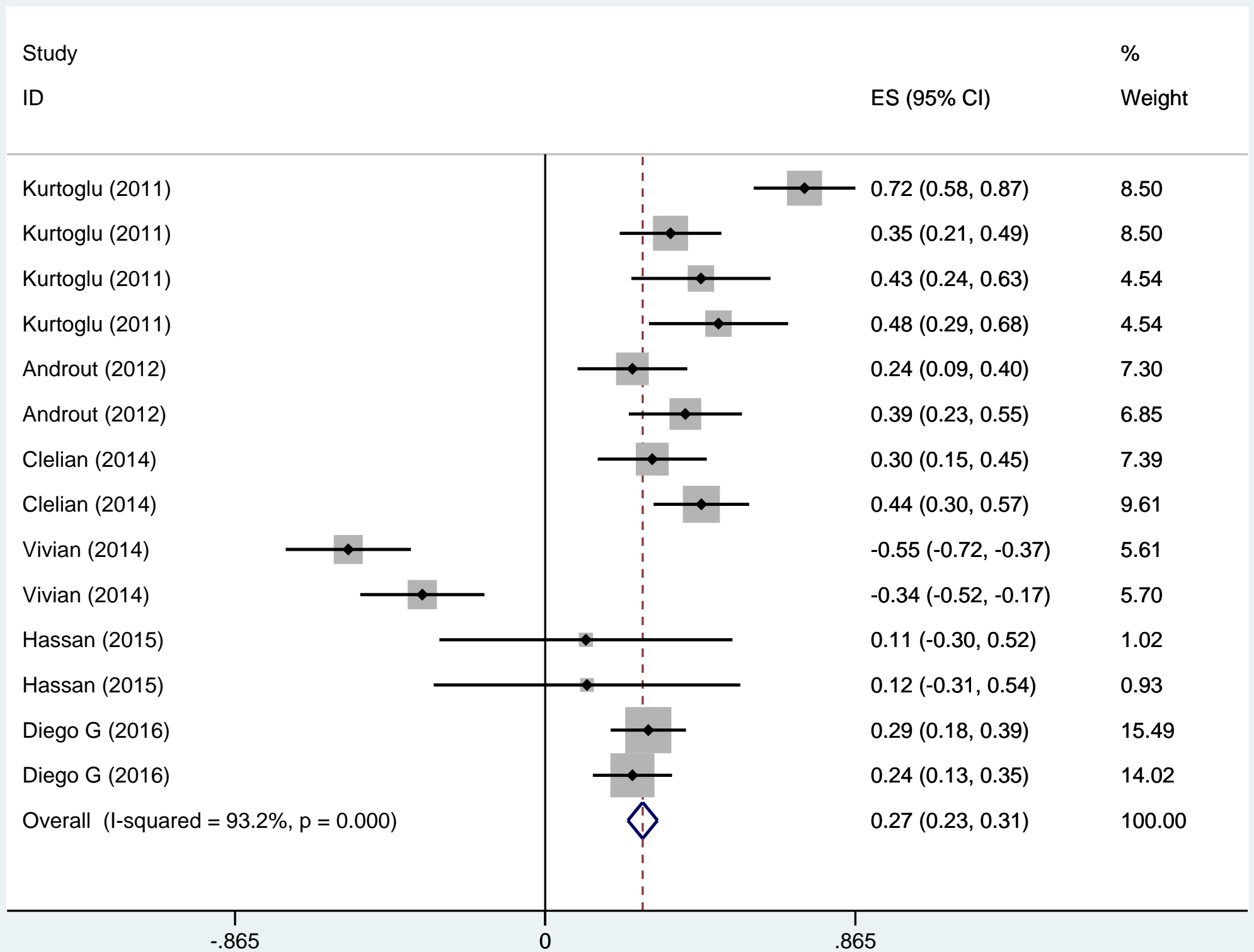

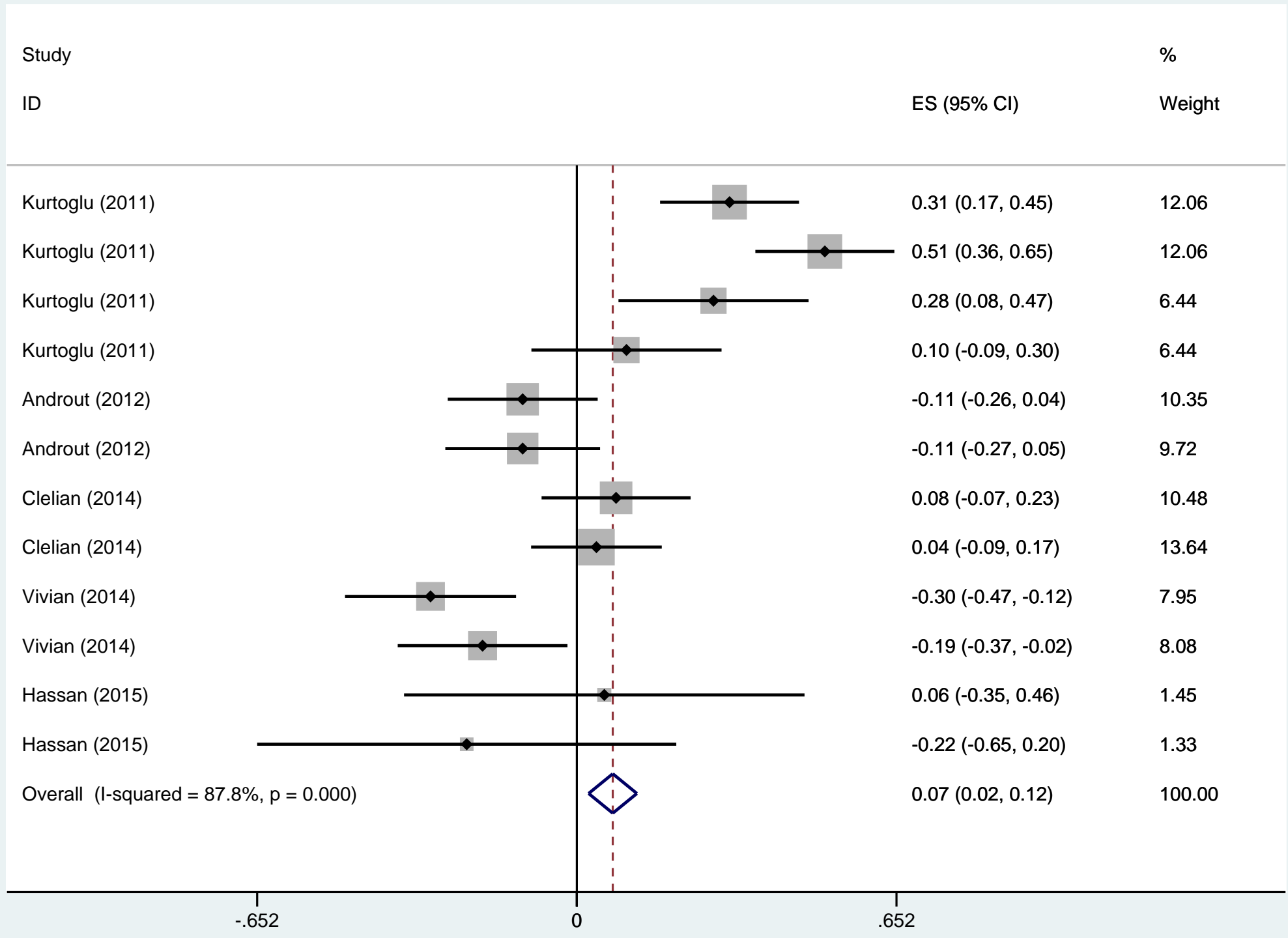

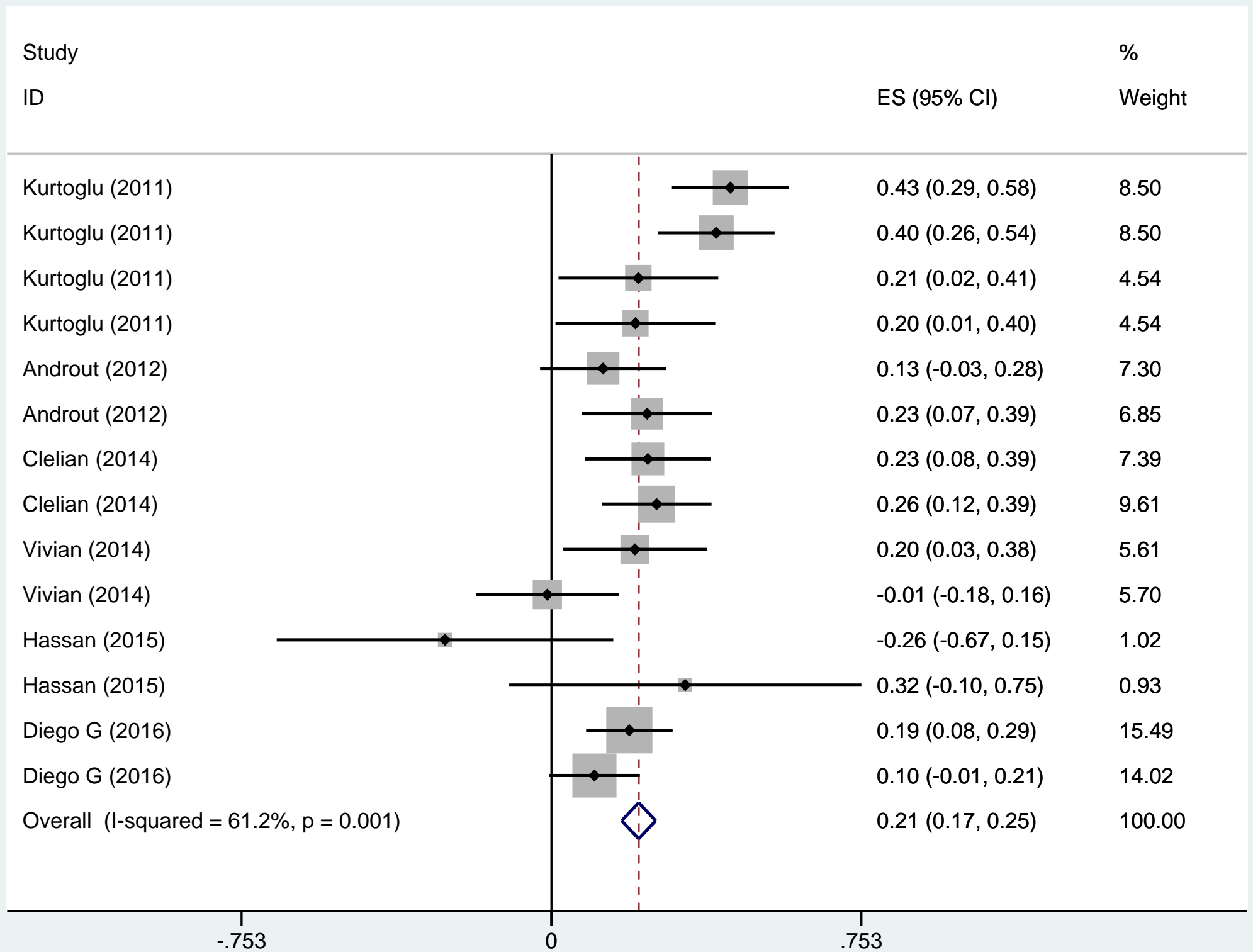

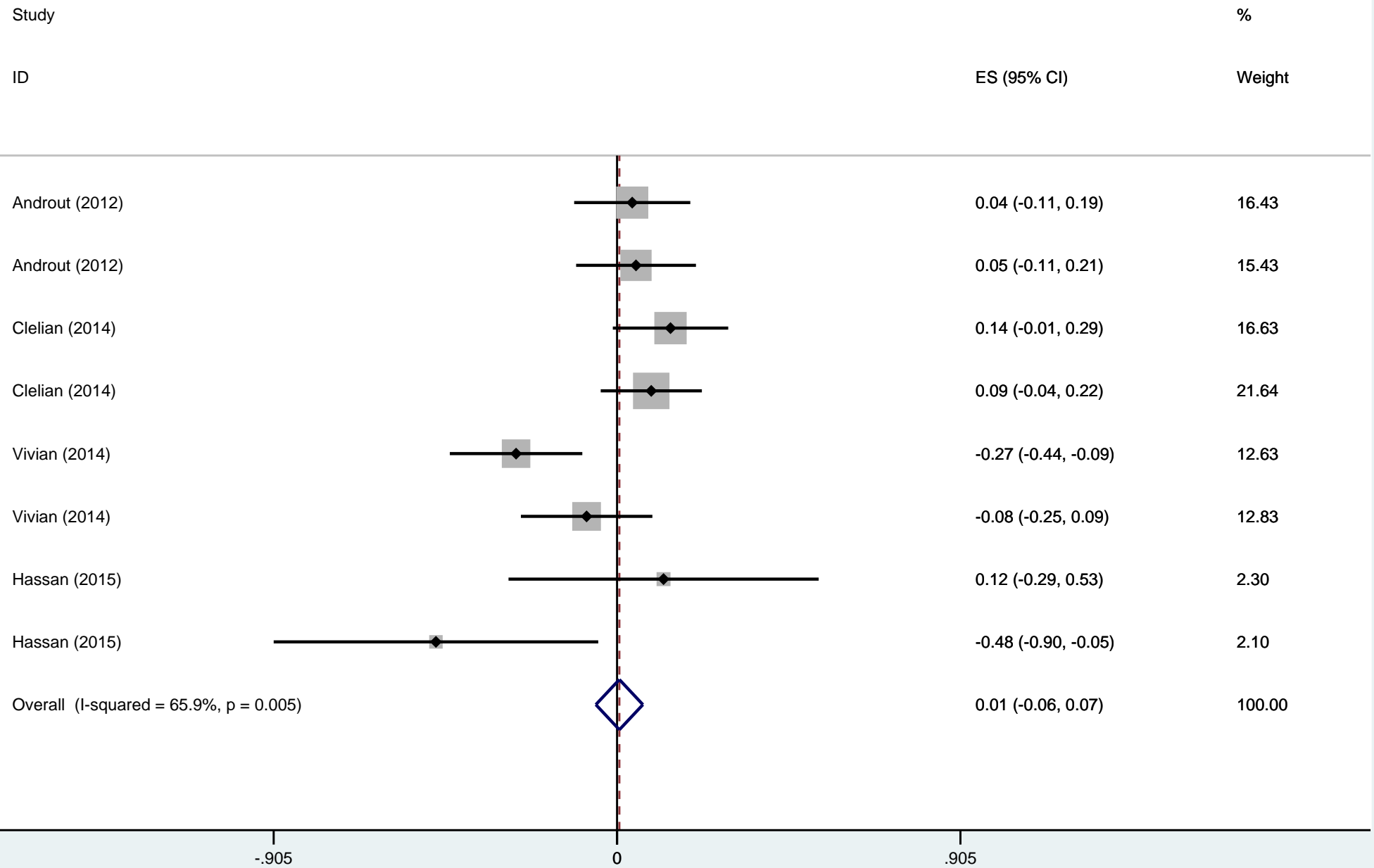

Supplement: Supplementary file 5 — Additional file 5: Figure S4. Forest plot of the association of neck circumference and 1) FBS, 2) HOMA, 3) TC, 4) TG, 5) LDL-C in child population. [file 13098_2018_373_MOESM5_ESM.pdf]
