# Supplementary material for: Neck circumference and its association with cardiometabolic risk factors: a systematic review and meta-analysis
Source: Diabetol Metab Syndr. 2018 Sep 29;10:72. doi: 10.1186/s13098-018-0373-y (PMC6162928; doi:10.1186/s13098-018-0373-y)
Supplement: Supplementary file 4 — Additional file 4: Table S1. Subgroup analysis for the association between neck circumference and cardio-metabolicfactors in adult population. [file 13098_2018_373_MOESM4_ESM.docx]

| **Table S1- Subgroup analysis for the association between neck circumference and cardio-metabolicfactors in adult population** | | | | |
| --- | --- | --- | --- | --- |
|  | No. effect size | Pooled effect size  (95% CI) | I^2^ (%) | P _heterogeneity_ |
| **TG** |  |  |  |  |
|  |  |  |  |  |
| ***Age***  Younger  older | 3  5 | 0.24 (0.20, 0.28)  0.23 (0.15,0.30) | 30.2  84.6 | 0.23  0.0001 |
| ***Sex***  Male  Female  Both | 3  3  2 | 0.27 (0.22,0.33)  0.22 (0.16, 0.28)  0.20(0.04, 0.36) | 62  59.1  88 | 0.07  0.08  0.004 |
| ***Location***  Asia  Non-Asia | 5  3 | 0.25 (0.19, 0.31)  0.21 (0.13,0.29) | 66.5  87.5 | 0.01  0.001 |
|  |  |  |  |  |
| **TC**  ***Sex*** |  |  |  |  |
| Male  Female | 2  2 | 0.16 (0.11, 0.22)  0.04 (-0.01, 0.08) | 18.7  0 | 0.26  0.62 |
| **FBS**  ***Sex*** |  |  |  |  |
| Male  Female | 3  3 | 0.18 (0.15, 0.22)  0.17 (0.11, 0.22) | 24  59.1 | 0.26  0.08 |
| **Location**  Asia  Non-Asia | 4  3 | 0.19 (0.16, 0.22)  0.13 (0.10, 0.16) | 0  28.3 | 0.61  0.24 |
|  |  |  |  |  |
|  |  |  |  |  |
